# Supplementary material for: Protocol for a feasibility study of OnTrack: a digital system for upper limb rehabilitation after stroke
Source: BMJ Open. 2020 Mar 23;10(3):e034936. doi: 10.1136/bmjopen-2019-034936 (PMC7103844; doi:10.1136/bmjopen-2019-034936)
Supplement: Author's manuscript [file bmjopen-2019-034936.draft_revisions.pdf]

BMJ Open is committed to open peer review. As part of this commitment we make the peer review history of every article we publish publicly available.

When an article is published we post the peer reviewers' comments and the authors' responses online. We also post the versions of the paper that were used during peer review. These are the versions that the peer review comments apply to.

The versions of the paper that follow are the versions that were submitted during the peer review process. They are not the versions of record or the final published versions. They should not be cited or distributed as the published version of this manuscript.

BMJ Open is an open access journal and the full, final, typeset and author-corrected version of record of the manuscript is available on our site with no access controls, subscription charges or pay-per-view fees (http://bmjopen.bmj.com).

If you have any questions on BMJ Open's open peer review process please email info.bmjopen@bmj.com

BMJ Open

Protocol for a feasibility study of OnTrack: a digital system for upper-limb rehabilitation after stroke.

Journal:	*BMJ Open*
Manuscript ID	bmjopen-2019-034936
Article Type:	Protocol
Date Submitted by the Author:	16-Oct-2019
Complete List of Authors:	Fusari, Gianpaolo; Royal College of Art, Helix Centre; Imperial College London, Helix Centre<br>Gibbs, Ella; Imperial College London, Helix Centre<br>Hoskin, Lily; Imperial College London, Helix Centre<br>Dickens, Daniel; Imperial College London, Helix Centre<br>Leis, Melanie; Imperial College London, Big Data and Analytical Unit, Institute of Global Health Innovation<br>Taylor, Elizabeth; Kingston and St George's Faculty of Health Social Care and Education<br>Jones, Fiona; Kingston and St George's Faculty of Health Social Care and Education<br>Darzi, Ara; Imperial College London, Helix Centre
Keywords:	Stroke < NEUROLOGY, REHABILITATION MEDICINE, NEUROLOGY, PUBLIC HEALTH

SCHOLARONE™
Manuscripts

I, the Submitting Author has the right to grant and does grant on behalf of all authors of the Work (as defined in the below author licence), an exclusive licence and/or a non-exclusive licence for contributions from authors who are: i) UK Crown employees; ii) where BMJ has agreed a CC-BY licence shall apply, and/or iii) in accordance with the terms applicable for US Federal Government officers or employees acting as part of their official duties; on a worldwide, perpetual, irrevocable, royalty-free basis to BMJ Publishing Group Ltd ("BMJ") its licensees and where the relevant Journal is co-owned by BMJ to the co-owners of the Journal, to publish the Work in this journal and any other BMJ products and to exploit all rights, as set out in our licence.

The Submitting Author accepts and understands that any supply made under these terms is made by BMJ to the Submitting Author unless you are acting as an employee on behalf of your employer or a postgraduate student of an affiliated institution which is paying any applicable article publishing charge ("APC") for Open Access articles. Where the Submitting Author wishes to make the Work available on an Open Access basis (and intends to pay the relevant APC), the terms of reuse of such Open Access shall be governed by a Creative Commons licence – details of these licences and which Creative Commons licence will apply to this Work are set out in our licence referred to above.

Other than as permitted in any relevant BMJ Author's Self Archiving Policies, I confirm this Work has not been accepted for publication elsewhere, is not being considered for publication elsewhere and does not duplicate material already published. I confirm all authors consent to publication of this Work and authorise the granting of this licence.

For peer review only

Title

Protocol for a feasibility study of OnTrack: a digital system for upper-limb rehabilitation after stroke.

Authors and affiliations

Gianpaolo Fusari[1], Ella Gibbs[1], Lily Hoskin[1], Daniel Dickens[1], Melanie Leis[2], Elizabeth Taylor[3], Fiona Jones[3], Ara Darzi[1]

[1]Helix Centre, Imperial College London and the Royal College of Art, London, United Kingdom
[2]Big Data and Analytical Unit, Institute of Global Health Innovation, Imperial College London, London, United Kingdom
[3]Kingston University and St George's, University of London, London, United Kingdom

Correspondence to:
Gianpaolo Fusari
HELIX Centre, Institute of Global Health Innovation
Imperial College London, 3rd Floor, Paterson Building, St Mary's Hospital, 16 South Wharf Road, London W2 1PF, UK
gianpaolo@helixcentre.com
+44 (0)77 6779 2770

Key words

Stroke, rehabilitation medicine, neurology, public health, digital health

Word count

3806

For peer review only

Abstract

Introduction

Arm weakness is a common problem after stroke (affecting 450,000 people in the UK) leading to loss of independence. Repetitive activity is critical for recovery but research shows people struggle with knowing what or how much to do, and keeping track of progress. Working with >100 therapists and patients, we co-developed the OnTrack intervention - consisting of software for smart-devices and coaching support - that has the potential to address this problem. This is a protocol to assess the feasibility of OnTrack for evaluation in a randomised control trial.

Methods and analysis

A mixed methods, single-arm study design will be used to evaluate the feasibility of OnTrack for hospital and community use. Participants from a stroke unit will be recruited (n=24) into the study and will be involved for 14 weeks. During week 1, 8 and 14 participants will complete assessments relating to their arm function, arm impairment, and activation. During weeks 2-13 participants will use OnTrack to track their arm movement in real time, receive motivational messages, and face-to-face sessions to address problems, gain feedback on activity, and receive self-management skills coaching. All equipment will be loaned to study participants. A parallel process evaluation will be conducted to assess the intervention's fidelity, dose and reach, using a mixed methods approach. A Public and Patient Involvement (PPI) group will oversee the study and help with interpretation of qualitative and quantitative data findings.

Ethics and dissemination

Ethical approval granted by the NHS Health Research Authority, Health and Care Research Wales, and the London - Surrey Research Ethics Committee (ref.19/LO/0881). Trial results will be submitted for publication in peer review journals, presented at international conferences and disseminated amongst stroke communities. The results of this trial will inform development of a definitive trial.

Trial registration details

ClinicalTrials.gov (NCT03944486), pre-results.

For peer review only

Strengths and limitations

- This is a feasibility trial of a novel intervention which employs an integrated approach for tracking arm activity and coaching with the aim of increasing stroke survivors' confidence and ability to use their impaired arm in daily activities, increasing the opportunities for repetitive rehabilitation.
- PPI involvement from more than 100 stroke survivors, carers, and clinicians have contributed to our needs-finding phase, co-designed OnTrack and informed the feasibility study. A new PPI group will oversee the running of the study and help with interpretation of qualitative and quantitative data findings.
- An independent process evaluation will provide detailed information about implementation, context, and the mechanisms of impact of the intervention. Findings will help in the understanding of intervention fidelity and training needs required for a definitive trial.
- For pragmatic reasons the study uses a non-randomised designed carried out at a single site- this will limit understanding about randomisation and recruitment
- Participants will not be followed-up after intervention; however participant views will be sought regarding appropriate follow-up times in a subsequent definitive trial.

For peer review only

Introduction

Every year around the world over 15 million people experience a stroke, leaving 5 million people with a permanent disability.(1) Stroke is the leading cause of disability in the UK; half of the nearly 1.2 million stroke survivors who live in the country have some form of disability, significantly contributing to the loss of independence and feeling of isolation that they experience. (2,3) Furthermore, stroke is estimated to cost UK society £26 billion every year, with the vast majority of these costs borne by the informal care sector.(2)

Upper-limb (arm) weakness is the main cause of physical impairment affecting 75% of disabled stroke survivors; this equates to around 450,000 people in the UK.(2) Dose-intensive repetitive rehabilitation is widely accepted as the 'gold-standard' for regaining ability after stroke, however, NHS resources are often limited and unable to provide this.(4,5) A recent Cochrane review of over 500 trials failed to yield high-quality practice recommendations.(6) Arm recovery after stroke is a national research priority,(7) nonetheless, studies suggest that the actual time patients spend exercising is minimal.(8,9) Many current approaches to solving this problem focus on improving the prescribed rehabilitation sessions, often employing gamification techniques.(10,11) Whilst this is important, there is untapped potential to increase repetitive rehabilitation by targeting the large proportion of the day where patients are going about their daily activities and can use their arm movement (however small) to a greater extent. Capacity for activity could be increased further by using self-management methods as demonstrated by several different programmes in stroke and other long-term conditions.(12-15) This has informed the

development of OnTrack which aims to increase opportunities for activity by improving individuals' self-management skills through tailored support and real-time activity feedback on their arm movement.

An ethnographic study conducted by the Helix Centre(16) (funded by Innovate UK) revealed that patients struggle to see and keep track of improvements, this impacts their motivation and leaves them dependent on therapists for feedback. Stroke survivors often report feeling unsupported after leaving hospital and not knowing how to best help themselves improve their arm function, confirming views documented by other studies.(17-19) Feedback gathered from over 100 stroke survivors and clinicians was the basis for developing the OnTrack intervention.

A proof-of-concept test of OnTrack gathered data from a small group of patients (n=7) and confirmed that the intervention was safe and generally users could understand how and when to use it. Participants reported they were more aware of their impaired arm and had increased confidence in using it for new tasks. A 20% mean increase in activity was observed. The work conducted to date suggests that OnTrack has the potential to be a scalable solution that requires minimal training and could be used in conjunction with NHS services to help increase the overall amount of arm rehabilitation received. This study will assess the feasibility of the OnTrack intervention and inform the design of a definitive randomised controlled trial (RCT) to evaluate its clinical effectiveness, and follows the Standard Protocol Items: Recommendations for Interventional Trials (SPIRIT) guidelines.(20)

Methods and analysis

Aims and objectives

The primary aim is to evaluate the feasibility of an RCT to test the effectiveness of the OnTrack intervention for upper limb rehabilitation after stroke.

The objectives are to:

- Assess the feasibility of recruitment from hyper-acute and acute stroke units, and rehabilitation wards to ascertain strategy and recruitment rates.
- Assess dropout rates by observing adherence and compliance with the intervention.
- Understand the acceptability and usability of the intervention by stroke survivors.
- Understand the acceptability of study procedures by healthcare professionals.
- Explore implementation fidelity, dose and reach of the OnTrack intervention.

The study will also collect clinical outcomes regarding arm function, impairment and activation to identify an appropriate primary outcome, and to estimate parameters for a sample size calculation for an RCT (Table 1).

For peer review only - http://bmjopen.bmj.com/site/about/guidelines.xhtml

Study design

A feasibility study with a nested process evaluation (Figure 1). The study is a single-site, non-randomised intervention trial. The design of the study was developed through a collaborative approach between the study researchers, a PPI steering group, front-line therapists, and the Research Design Service at the National Institute for Health Research.

An independent process evaluation will be conducted in parallel to learn about usage and engagement mechanisms of participants, therapists and other frontline staff, providing critical information for implementation fidelity and impact mechanisms necessary for scale-up.

Study setting

The study will be conducted at an inner city NHS hospital Trust in London. Recruited participants will be able to continue to receive the intervention at home if discharged from hospital prior to ending the intervention period (14 weeks).

Participants

The inclusion criteria encompasses:

- Adults (aged 18 or over).
- Stroke diagnosis less than 6 months previously (first or recurrent).
- Arm impairment of any type or level (including weakness - including dense hemiplegia, neglect, and sensory deficits).
- Ability to provide informed consent.
- Reliability to communicate (verbally or nonverbally) and understand English.
- Ability to read a predefined short message.

Potential participants who at the time of recruitment present with any of the following will be excluded:

- Unstable medical condition.
- Severe pain in the arm affected either at rest or during movement.
- Severe oedema in the arm affected by their stroke.
- Known discharge plans to a hospital other than the site Trust or residential care in less than 7 weeks.

Recruitment

Participants will be recruited from the Hyperacute Stroke Unit (HASU), Acute Stroke Unit (ASU), and Clinical Neurorehabilitation Unit (CNRU) at an inner city NHS hospital Trust in London.

Stroke clinicians will be responsible for screening and identifying suitable patients. They will introduce the study to potential participants and provide information documents. Potential

For peer review only

participants will be given a minimum of 24 hours to consider the advantages and disadvantages of participating in the study and to formulate questions. Therapists will be able to answer questions or will liaise with the research team to provide an answer. Once all questions are answered and a potential participant is willing to participate, consent will be taken by the therapist. Only at this stage will patient information be shared with the research team. There may be situations where a therapist is only able to take verbal consent from a participant due to time or material constraints, in such cases the researchers will be able to take written consent from the participant upon first meeting them.

Sample size calculation

Guidelines advocate a sample size of 12-30 participants for feasibility studies.(21) Experienced clinical academics and clinicians at the trial site have advised to expect about 50% of eligible patients to agree to participation and a 50% completion rate. This has informed a recruitment plan to identify at least 60 potential participants in a period of 30 weeks to reach the minimum sample size.

Intervention

The OnTrack system consists of smart-devices (smartphone and smartwatch), software (OnTrack app), and coaching support. Smart-devices are used to track arm movement. Motivational messages and a real-time display of completed arm activity are presented to the user via the OnTrack app. Coaching support is provided through fortnightly consultations by the researchers. During consultations, participants will receive self-management training informed by the Bridges(22) and TaCAS(23) self-management programs. Data gathered by the OnTrack system can be accessed by the researchers via a digital dashboard to inform consultations.

Participants will be loaned all equipment necessary for the trial and no previous experience with using smart-devices is required to participate. Technical support will be provided only in cases where the hardware and/or software fail to perform the required functions to deliver the intervention.

Table 2 provides a participation schedule and a summary of the intervention procedures.

Outcomes

Feasibility of trial design and procedures

- Recruitment strategy and rates (feasibility of recruitment from HASU, ASU, CNRU wards) - percentage of patients: screened; eligible; approached; consented; excluded after screening. Participants consented and recruited will be logged in DOCUMAS(24)
- Compliance and adherence to intervention - percentage of participants who start OnTrack daily for the duration of the intervention period, measure of minutes of activity

For peer review only

per participant as recorded by the OnTrack app, engagement with OnTrack app as measured by system analytics.

- Completion rates - percentage of participants who complete the intervention
- Acceptability and reasons for decline/withdrawal - number of participants who withdraw or decline the intervention and reasons why

Clinical assessments

As a secondary objective, clinical outcomes will be collected at different time points to identify an appropriate primary outcome, and to estimate parameters for a sample size calculation for an RCT (Table 1). The outcome measures and assessments are listed below.

Patient activation

Patient activation is a concept recognised by the NHS that describes the knowledge, skills and confidence a person has in managing their own health and health care.(25) This will be measured using the Patient Activation Measure (PAM)(26) which has been validated in stroke populations in the UK.(27) The PAM survey measures patients on a 0–100 scale and can categorise patients into one of four activation levels along an empirically derived continuum.(26) Activation levels will be used to allocate participants one of three different OnTrack coaching tiers.

Arm impairment

Arm impairment will be measured objectively using the Fugl-Meyer Assessment for upper extremity (FMA-UE).(28) The FMA-UE has been tested extensively, and is found to have excellent psychometric properties and is recommended as core measures to be used in every stroke recovery and rehabilitation trial.(29)

Arm function

Arm function will be assessed using the Upper-Extremity Motor Activity Log-14 (MAL).(30) The MAL is a scripted, structured interview developed to self-report the amount and quality of use of the impaired arm in individuals with stroke in 14 different activities of daily living.

Gross level of disability

The modified Rankin Scale (mRS)(31) is the most prevalent functional outcome measure in contemporary stroke trials. The mRS quantifies disability using an ordinal hierarchical grading from zero (no symptoms) to 5 (severe disability).

Arm pain

Pain will be assessed using a visual analogue scale (VAS) from 0 (no pain) to 10 (excruciating pain) over the last 24 hours. VAS is a valid measure of pain intensity and is responsive to change.(32) Individuals scoring 3/10 or more in the affected arm will be excluded/withdrawn from the study unless their pain is only on movements that are not part of their usual everyday activities (e.g. arm pain when doing overhead reaching).

For peer review only

Cognitive impairment

Cognitive impairment will be assessed using the Montreal Cognitive Assessment (MoCA). The MoCA is a brief cognitive screening tool with high sensitivity and specificity for detecting mild cognitive impairment.(33)

Perceptual neglect

Albert's Test (AT) is a simple test where participants are asked to cross out lines ruled in a standard fashion on a sheet of paper. The test is very easy to administer and is a good predictor of functional activity six months after stroke onset.(34)

Quality of life

The EQ-5D-5L is a widely used standardised preference based measure of health status developed by the EuroQol Group in order to provide a simple, generic measure of health for clinical and economic appraisal.(35)

Additional assessments

A Lap-to-Table (LTT) timed test will be performed where the researchers measure the time it takes a participant to move their hand three times from resting on their lap to a table positioned in front of them. This test is performed to assess its potential to use as part of the inclusion criteria for an RCT.

The NHS Friends and Family Test (FFT)(36) will be used to obtain feedback on the overall experience of using OnTrack and participating in the trial. Participants will be asked: "How likely are you to recommend OnTrack to friends and family if they needed similar care or treatment?" with answers provided in a Likert 5-point scale ranging from "extremely likely" to "extremely unlikely" and an "I don't know" option.

The System Usability Scale (SUS) will be used to subjectively assess the usability of the OnTrack intervention. The test is a simple, ten-item scale covering a variety of aspects of system usability, such as the need for support, training, and complexity, and thus have a high level of face validity for measuring the usability of a system.(37)

Process evaluation

A process evaluation will be carried out by researchers working independently to the intervention team and in parallel to the trial to determine whether the OnTrack intervention was delivered as intended and to understand the mechanisms of impact. The aim of the process evaluation at the feasibility stage is mainly to understand how the trial design and intervention could be optimised ahead of an RCT.(38) A logic model(39,40)) that defines the intervention in terms of inputs, outputs, causal assumptions and expected outcomes has been developed to help identify core questions for the evaluation team to explore (Figure 2). The evaluation team will observe a percentage of all intervention sessions with the objective of documenting fidelity, dose and reach of the intervention.

For peer review only

Interim results will be shared with the intervention team at the half-way point with the objective to review some of the procedures and make minor adjustments as necessary.

In-depth semi-structured interviews will be conducted with patients at the end of their participation, a minimum sample of 12 is anticipated. A topic guide with themes drawing from the logic model will be used. Interviews will focus on participants' experiences using OnTrack, their perceptions of arm tracking, motivational messaging and the researcher consultations. Additionally, the interviews will explore participants' perceptions of the impact OnTrack had on them in terms of progress, awareness, participation, and confidence in self-management. Participants' responses will be compared against activity data collected from the OnTrack app.

NHS therapists caring for participants taking part will be invited to complete a short online survey to gather their feedback regarding acceptability of study procedures.

Data analysis

Analysis will be completed on the parameters and implementation of the study in addition to the usability of OnTrack.

Data collected for the process evaluation will capture changes over time and will be a combination of qualitative data from interviews with stroke participants and therapists to explore their experiences of using OnTrack, as well as quantitative data on usage of OnTrack and the self-reported SUS. OnTrack therapy support sessions will be monitored through a fidelity checklist and observations. Interview data will undergo thematic analysis by the evaluation team. Data will be entered into NVIVO,(41) line by line coding and analysis will be informed by Braun and Clark's approach to thematic analysis.(42)

The team will analyse users' activity patterns by day and hour of day. This will allow an understanding of how usage varies for each user over time, as well as how patterns of usage vary from one user to another. Figure 3 includes examples of visualisations created using aggregated data captured by OnTrack from beta testers (all healthy individuals) between June-August 2019. By adding self-reported SUS data to the analyses, the team will be able to explore the potential correlation between SUS and OnTrack usage.

Subgroup analyses are planned based on patient demographics, stroke disability, stroke subtype and the care pathway patients go through during the intervention period.

All data will be stored and accessed in accordance with GDPR guidance.

Clinical trial support will be provided by the Big Data and Analytical Unit (BDAU) at Imperial College London's Institute of Global Health Innovation (IGHI).

For peer review only

Patient and public involvement (PPI)

To date, over 100 stroke survivors, carers and therapists have been involved in the design of OnTrack. Participants and have been instrumental in highlighting areas for improvement in upper-limb stroke rehabilitation. They have contributed to a co-design process (including workshops, interviews, observations and surveys) resulting in the design, development and initial testing of OnTrack.

A steering group comprising of four stroke survivors was formed for the purpose of this feasibility study. Diversity within the group - both in terms of demographics and stroke severity - was considered. The group has supervised the development of all patient-facing material ensuring its clarity. They will also participate in data analysis by helping to refine themes and key messages arising from qualitative interviews. Participants will be trained by experienced researchers for this purpose.

The steering group will meet five times over the duration of the study, including an initial briefing session at the start to outline their involvement. Steering group members will be key members of the research team and their time and travel will be reimbursed according to INVOLVE(43) guidelines.

The PPI involvement plan was shared with Imperial College London's PPI 'Research Partners Group' on 21.02.19 who felt that the needs of the steering group have been accounted for.

Ethics and dissemination

The OnTrack study will be conducted in accordance with the recommendations for physicians involved in research on human subjects adopted by the 18th World Medical Assembly, Helsinki 1964 and later revisions; and in compliance with the relevant UK and European legislation including the NHS Health Research Authority (HRA) policy frameworks and the General Data Protection Regulation 2018 (GDPR).

The study was granted ethical approval by the HRA, Health and Care Research Wales, and the London - Surrey Research Ethics Committee (ref. 19/LO/0881). Local site capacity and capability approval has been granted by the hospital Trust.

The current approved protocol version is V1.3 dated 19.06.2019. Protocol amendments will be submitted for approval to the NHS HRA in the first instance and to the local site thereafter ahead of implementation.

The Chief Investigator is responsible for preserving the confidentiality of participants taking part in the study. Researchers will have patients' names, contact numbers, emails and home addresses for the purposes of arranging visits. This information will be stored in accordance with GDPR legislation. Participants are free to withdraw from the study at any time. However, anonymised activity data collected may still be used for data analysis as this is unlinked of any patient identifiable information.

For peer review only

The day-to-day management of the study will be coordinated by the Helix Centre. A study steering committee formed by the intervention team, evaluation team, PPI group, and representatives from the local site will meet at regular intervals throughout the study.

Regular updates about the trial will be made available through social media, blog posts, newsletters and the Helix Centre website (www.helixcentre.com). Trial results will be submitted for publication in journals, presented at national and international stroke meetings and conferences and disseminated amongst stroke communities.

Trial status

The first participant was enrolled on 09.09.2019 and recruitment is expected to complete by the end of March 2020. Enrolment and data collection was continuing as planned at the time of submission of this protocol.

Author statement

AD is grant holder and has project oversight along with DD. GF, EG and LH developed the intervention and conceived of the study. GF, EG, and FJ initiated the study design and ET and ML helped with further refinement. EG and GF are responsible for delivering the intervention and data collection. FJ and ET are responsible for the process evaluation. ML provides statistical expertise in trial design and is conducting the primary statistical analysis. All authors contributed to the refinement of the study protocol and approved the final manuscript.

Funding

This study is funded by the NIHR Imperial Biomedical Research Centre (BRC), grant 1215-20013. The views expressed are those of the author(s) and not necessarily those of the NIHR or the Department of Health and Social Care.

Data statement

Anonymised data will be made available in a public repository once the data have obtained validation through publication.

Conflict of interests

FJ is the founder of the social enterprise Bridges Self-Management. She has not received any financial support for this work that could have influenced the design.

For peer review only

Acknowledgments

The authors would like to acknowledge the contributions of the members of the PPI group; Jennifer Crow and Sarah Daniels for their input on recruitment and sample size calculations; and Dr Gaby Judah for her help in defining some aspects of the intervention.

Figure legends

Figure 1

Trial diagram

Figure 2

Logic model

Figure 3

Examples of visualisations created using aggregated data captured by OnTrack from healthy beta testers between June-August 2019

For peer review only

Table 1

Outcome measures

Concept	Assessment	Week of administration
Patient Activation / Engagement	Patient Activation Measure (PAM)	1, 8, 14
Arm impairment	Fugl-Meyer Assessment for upper extremity (FMA-UE)	1, 8, 14
Arm function	Upper-Extremity Motor Activity Log-14 (MAL)	1, 8, 14
Gross level of disability	modified Rankin Scale (mRS)	1, 8, 14
Arm pain	Visual Analogue Scale (VAS)	1, 8, 14
Cognitive impairment	Montreal Cognitive Assessment (MoCA)	1, 8, 14
Arm neglect	Albert's Test (AT)	1, 8, 14
Quality of life	EQ-5D-5L	1, 8, 14
Arm function	Lap-to-Table (LTT)	1, 8, 14
Service experience	Friends and Family Test (FTT)	8, 14
System usability	System Usability Scale (SUS)	14

For peer review only

Table 2

Participation schedule

Week	Phase	Description	OnTrack consultation	Assessments
0	Information and consent	NHS therapists screen for eligible patients, provide information and consent participants		Screening, information, and consent
1	Baseline assessment (initial)	Participants complete outcome measures and wear activity trackers (Axivity AX3) on both arms for one week to gather a baseline of activity allowing left-to-right usage comparison		PAM, FMA-UE, MAL, mRS, VAS, MoCA, AT, EQ-5D-5L, LTT
2	OnTrack intervention	Participants wear a smartwatch (Apple Watch Series 3 or 4) on their impaired arm only. They will receive real-time feedback on the amount of movement completed (measured in minutes) and daily motivational messages. Participants will receive fortnightly consultations with a researcher to troubleshoot and receive self-management skills training<br><br>Baseline assessments are repeated during week 8 (halfway)	Onboarding	
3			Check-in & self-management skills training (Problem Solving)	
4				
5			Check-in & self-management skills training (Self-Discovery)	
6				
7			Check-in & self-management skills training (Goal Setting)	
8			Halfway assessment<br><br>Check-in & self-management skills training (Goal Setting cont.)	PAM, FMA-UE, MAL, mRS, VAS, MoCA, AT, EQ-5D-5L, LTT, FFT
9				

For peer review only

10			Check-in & self-management skills training (Reflection)	
11				
12			Check-in & self-management skills training (Sign-posting)	
13				
14	Baseline assessment (exit)	Participants complete outcome measures and wear activity trackers (Axivity AX3) on both arms for one week to gather a baseline of activity allowing left-to-right usage comparison		PAM, FMA-UE, MAL, mRS, VAS, MoCA, AT, EQ-5D-5L, LTT, FFT, SUS
15	Feedback	Independent evaluator leads feedback sessions with participants who have completed the intervention<br><br>End of participation		Semi-structured interview, online survey (therapists)

References

(1) WHO. *The Atlas of Heart Disease and Stroke.* Available from: https://www.who.int/cardiovascular_diseases/resources/atlas/en/ [Accessed 04 Oct 2019].
(2) Stroke Association. *State of the Nation: stroke statistics.* Stroke Association. 2018.
(3) Salter K, Hellings C, Foley N, Teasell R. The Experience of Living with Stroke: A Qualitative Meta-Synthesis. *Journal of Rehabilitation Medicine.* 2008; 40 (8): 595-602. Available from: doi: 10.2340/16501977-0238 Available from: http://www.ingentaconnect.com/content/mjl/sreh/2008/00000040/00000008/art00003 .
(4) Pollock A, Farmer SE, Brady MC, Langhorne P, Mead GE, Mehrholz J, et al. Interventions for improving upper limb function after stroke. *The Cochrane database of systematic reviews.* 2014; 2014 (11): CD010820. Available from: doi: 10.1002/14651858.CD010820.pub2 Available from: https://www.ncbi.nlm.nih.gov/pubmed/25387001 .
(5) Care Quality Commission. *Supporting life after stroke: a review of services for people who have had a stroke and their carers.* London: Care Quality Commission. 2011.
(6) Van Peppen RP, Kwakkel G, Wood-Dauphinee S, Hendriks HJ, Van der Wees, Ph J, Dekker J. The impact of physical therapy on functional outcomes after stroke: what's the evidence? *Clinical Rehabilitation.* 2004; 18 (8): 833-862. Available from: doi: 10.1191/0269215504cr843oa Available from: https://journals.sagepub.com/doi/full/10.1191/0269215504cr843oa .
(7) Pollock A, St George B, Fenton M, Firkins L. Top 10 Research Priorities Relating to Life after

Stroke – Consensus from Stroke Survivors, Caregivers, and Health Professionals. *International Journal of Stroke.* 2014; 9 (3): 313-320. Available from: doi: 10.1111/j.1747-4949.2012.00942.x Available from: https://journals.sagepub.com/doi/full/10.1111/j.1747-4949.2012.00942.x .
(8) Kunkel D, Fitton C, Burnett M, Ashburn A. Physical inactivity post-stroke: a 3-year longitudinal study. *Disability & Rehabilitation.* 2015; 37 (4): 304-310. Available from: doi: 10.3109/09638288.2014.918190 Available from: http://www.tandfonline.com/doi/abs/10.3109/09638288.2014.918190 .
(9) Bernhardt J, Dewey H, Thrift A, Donnan G. Inactive and Alone: Physical Activity Within the First 14 Days of Acute Stroke Unit Care. *Stroke.* 2004; 35 (4): 1005-1009. Available from: doi: 10.1161/01.STR.0000120727.40792.40 Available from: http://stroke.ahajournals.org/cgi/content/abstract/35/4/1005 .
(10) Laver KE, Lange B, George S, Deutsch JE, Saposnik G, Crotty M. Virtual reality for stroke rehabilitation. *The Cochrane database of systematic reviews.* 2017; 11 (11): CD008349. Available from: doi: 10.1002/14651858.CD008349.pub4 Available from: https://www.ncbi.nlm.nih.gov/pubmed/29156493 .
(11) Loureiro R, Harwin W, Nagai K, Johnson M. Advances in upper limb stroke rehabilitation: a technology push. *Medical & Biological Engineering & Computing.* 2011; 49 (10): 1103-1118. Available from: doi: 10.1007/s11517-011-0797-0 Available from: https://www.ncbi.nlm.nih.gov/pubmed/21773806 .
(12) Jones F, Gage H, Drummond A, Bhalla A, Grant R, Lennon S, et al. Feasibility study of an integrated stroke self-management programme: a cluster-randomised controlled trial. *BMJ Open.* 2016; 6 (1): e008900. Available from: doi: 10.1136/bmjopen-2015-008900 Available from: http://dx.doi.org/10.1136/bmjopen-2015-008900 .
(13) Harwood M, Weatherall M, Talemaitoga A, Barber PA, Gommans J, Taylor W, et al. Taking charge after stroke: promoting self-directed rehabilitation to improve quality of life – a randomized controlled trial. *Clinical Rehabilitation.* 2012; 26 (6): 493-501. Available from: doi: 10.1177/0269215511426017 Available from: https://journals.sagepub.com/doi/full/10.1177/0269215511426017 .
(14) Kendall E, Catalano T, Kuipers P, Posner N, Buys N, Charker J. Recovery following stroke: The role of self-management education. *Social Science & Medicine.* 2007; 64 (3): 735-746. Available from: doi: 10.1016/j.socscimed.2006.09.012 Available from: https://www.sciencedirect.com/science/article/pii/S0277953606004801 .
(15) Grady PA, Gough LL. Self-Management: A Comprehensive Approach to Management of Chronic Conditions. *American journal of public health.* 2014; 104 (8): e25-e31. Available from: doi: 10.2105/AJPH.2014.302041 Available from: https://www.ncbi.nlm.nih.gov/pubmed/24922170 .
(16) Helix Centre. *Project OnTrack.* Available from: https://helixcentre.com/project-ontrack [Accessed 04 Oct 2019].
(17) Buckland D. Leaving hospital is 'like falling off a cliff' for stroke survivors. *Raconteur.* APRIL 27, 2017 . Available from: https://www.raconteur.net/healthcare/leaving-hospital-is-like-falling-off-a-cliff-for-stroke-survivors [Accessed Oct 04 2019].
(18) McKevitt C, Fudge N, Redfern J, Sheldenkar A, Crichton S, Rudd AR, et al. Self-Reported Long-Term Needs After Stroke. *Stroke.* 2011; 42 (5): 1398-1403. Available from: doi: 10.1161/STROKEAHA.110.598839 Available from: https://www.ncbi.nlm.nih.gov/pubmed/21441153 .
(19) Woodman P, Riazi A, Pereira C, Jones F. Social participation post stroke: a meta-ethnographic review of the experiences and views of community-dwelling stroke survivors. *Disability & Rehabilitation.* 2014; 36 (24): 2031-2043. Available from: doi: 10.3109/09638288.2014.887796 Available from: http://www.tandfonline.com/doi/abs/10.3109/09638288.2014.887796 .
(20) Chan A, Tetzlaff JM, Altman DG, Laupacis A, Gøtzsche PC, Krleža-Jerić K, et al. SPIRIT

For peer review only

2013 Statement: Defining Standard Protocol Items for Clinical Trials. *Annals of internal medicine.* 2013; 158 (3): 200-207. Available from: doi: 10.7326/0003-4819-158-3-201302050-00583 Available from: https://www.ncbi.nlm.nih.gov/pubmed/23295957 .
(21) Billingham SAM, Whitehead AL, Julious SA. An audit of sample sizes for pilot and feasibility trials being undertaken in the United Kingdom registered in the United Kingdom Clinical Research Network database. *BMC medical research methodology.* 2013; 13 (1): 104. Available from: doi: 10.1186/1471-2288-13-104 Available from: https://www.ncbi.nlm.nih.gov/pubmed/23961782 .
(22) Bridges Self-Management Limited. *Bridges Self-Management.* Available from: http://www.bridgesselfmanagement.org.uk/ [Accessed 07 Oct 2019].
(23) Medical Research Institute of New Zealand. *Taking Charge After Stroke (TaCAS).* Available from: https://mrinz.ac.nz/programmes/stroke/ [Accessed 07 oct 2019].
(24) DOCUMAS. *DOCUMAS: efficient health research management.* Available from: https://www.documas.eu/ [Accessed 04 Oct 2019].
(25) Hibbard J, Gilburt H. *Supporting people to manage their health An introduction to patient activation.* London: The Kings Fund. 2014.
(26) Hibbard JH, Stockard J, Mahoney ER, Tusler M. Development of the Patient Activation Measure (PAM): Conceptualizing and Measuring Activation in Patients and Consumers. *Health Services Research.* 2004; 39 (4p1): 1005-1026. Available from: doi: 10.1111/j.1475-6773.2004.00269.x Available from: https://onlinelibrary.wiley.com/doi/abs/10.1111/j.1475-6773.2004.00269.x .
(27) Roberts NJ, Kidd L, Dougall N, Patel IS, McNarry S, Nixon C. Measuring patient activation: The utility of the Patient Activation Measure within a UK context—Results from four exemplar studies and potential future applications. *Patient Education and Counseling.* 2016; 99 (10): 1739-1746. Available from: doi: 10.1016/j.pec.2016.05.006 Available from: https://www.clinicalkey.es/playcontent/1-s2.0-S0738399116301938 .
(28) Sahlgrenska Academy Institute of Neuroscience and Physiology, University of Gothenburg. *Fugl-Meyer.* Available from: https://neurophys.gu.se/english/departments/clinical_neuroscience_and_rehabilitation/rehabilitation-medicine/fugl-meyer [Accessed 04 Oct 2019].
(29) Pamela W Duncan, Martha Propst, Steven G Nelson. Reliability of the Fugl-Meyer Assessment of Sensorimotor Recovery Following Cerebrovascular Accident. *Physical Therapy.* 1983; 63 (10): 1606-1610. Available from: doi: 10.1093/ptj/63.10.1606 Available from: http://ptjournal.apta.org/content/63/10/1606.abstract .
(30) Uswatte G, Taub E, Morris D, Vignolo M, McCulloch K. Reliability and Validity of the Upper-Extremity Motor Activity Log-14 for Measuring Real-World Arm Use. *Stroke.* 2005; 36 (11): 2493-2496. Available from: doi: 10.1161/01.STR.0000185928.90848.2e Available from: http://stroke.ahajournals.org/cgi/content/abstract/36/11/2493 .
(31) Quinn TJ, Dawson J, Walters MR, Lees KR. Exploring the Reliability of the Modified Rankin Scale. *Stroke.* 2009; 40 (3): 762-766. Available from: doi: 10.1161/STROKEAHA.108.522516 Available from: http://stroke.ahajournals.org/cgi/content/abstract/40/3/762 .
(32) Jensen MP, Karoly P, Braver S. The measurement of clinical pain intensity: a comparison of six methods. *Pain.* 1986; 27 (1): 117-126. Available from: doi: 10.1016/0304-3959(86)90228-9 Available from: https://www.sciencedirect.com/science/article/pii/0304395986902289 .
(33) Nasreddine ZS, Phillips NA, Bédirian V, Charbonneau S, Whitehead V, Collin I, et al. The Montreal Cognitive Assessment, MoCA: A Brief Screening Tool For Mild Cognitive Impairment. *Journal of the American Geriatrics Society.* 2005; 53 (4): 695-699. Available from: doi: 10.1111/j.1532-5415.2005.53221.x Available from: https://onlinelibrary.wiley.com/doi/abs/10.1111/j.1532-5415.2005.53221.x .
(34) Fullerton KJ, McSherry D, Stout RW. Albert's test: a neglected test of perceptual neglect. *Lancet (London, England).* 1986; 1 (8478): 430. Available from:

https://www.ncbi.nlm.nih.gov/pubmed/2868349 .
(35) EuroQol - a new facility for the measurement of health-related quality of life. *Health policy.* 1990; 16 (3): 199-208. Available from: doi: 10.1016/0168-8510(90)90421-9 Available from: https://www.sciencedirect.com/science/article/pii/0168851090904219 .
(36) NHS England. *The Friends and Family Test.* NHS England. 2014.
(37) SUS: A 'Quick and Dirty' Usability Scale. *Usability Evaluation In Industry.* CRC Press; 1996. pp. 207-212.
(38) Moore GF, Audrey S, Barker M, Bond L, Bonell C, Hardeman W, et al. Process evaluation of complex interventions: Medical Research Council guidance. *BMJ : British Medical Journal.* 2015; 350 (mar19 6): h1258. Available from: doi: 10.1136/bmj.h1258 Available from: http://dx.doi.org/10.1136/bmj.h1258 .
(39) W.K. Kellogg Foundation. *Logic Model Development Guide.* Battle Creek, Michigan: W.K. Kellogg Foundation. 2004.
(40) The Scottish Government. *Designing and Evaluating Behaviour Change Interventions.* The Scottish Government. 2015.
(41) QSR International Ltd. *NVIVO.* Available from: https://www.qsrinternational.com/nvivo/nvivo-products [Accessed 07 Oct 2019].
(42) Braun V, Clarke V. Using thematic analysis in psychology. *Qualitative Research in Psychology.* 2006; 3 (2): 77-101. Available from: doi: 10.1191/1478088706qp063oa Available from: http://www.tandfonline.com/doi/abs/10.1191/1478088706qp063oa .
(43) National Institute for Health Research. *About INVOLVE.* Available from: https://www.invo.org.uk/about-involve/ [Accessed 04 Oct 2019].

For peer review only

Figure 1

Trial diagram

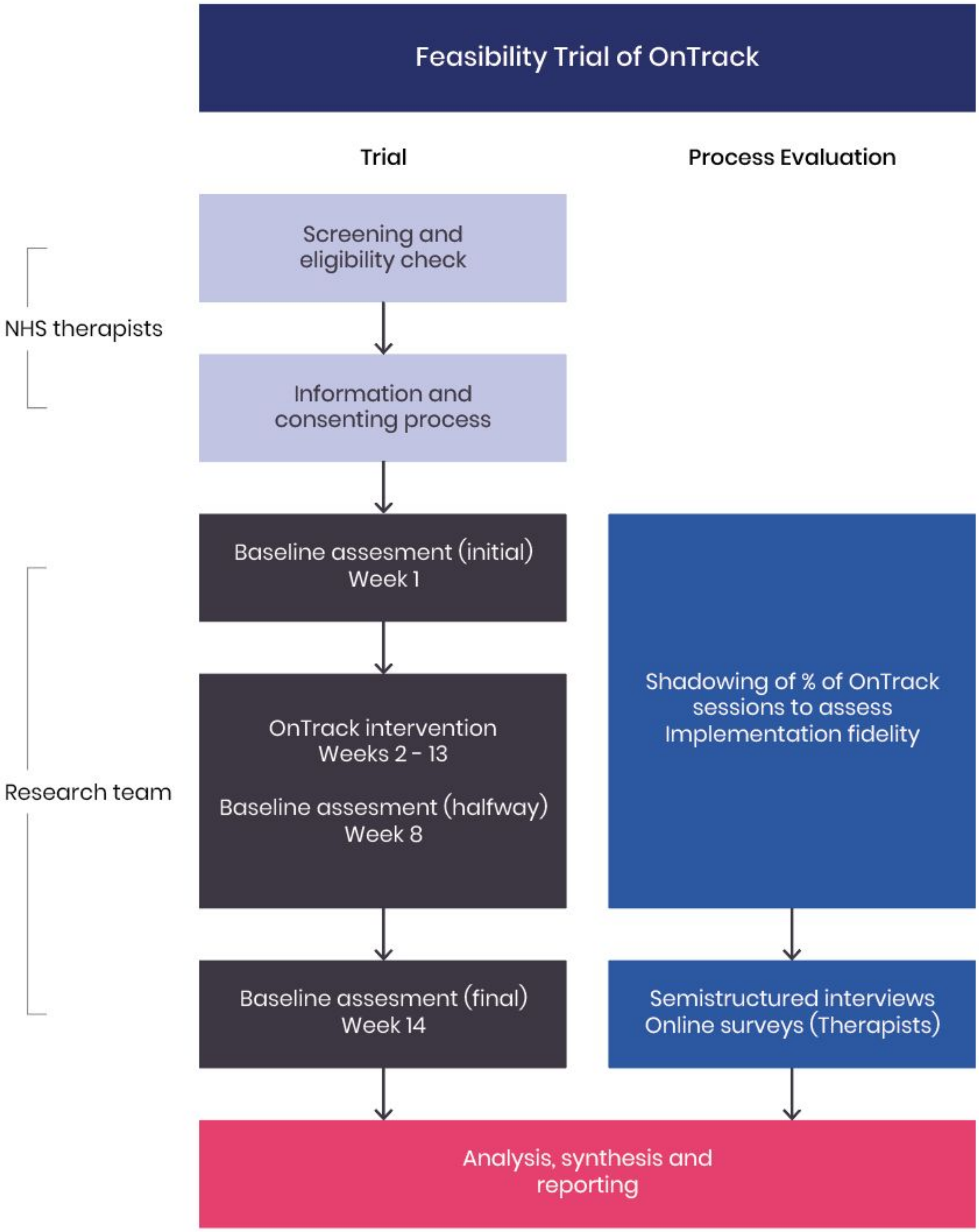

Figure 2

Logic model

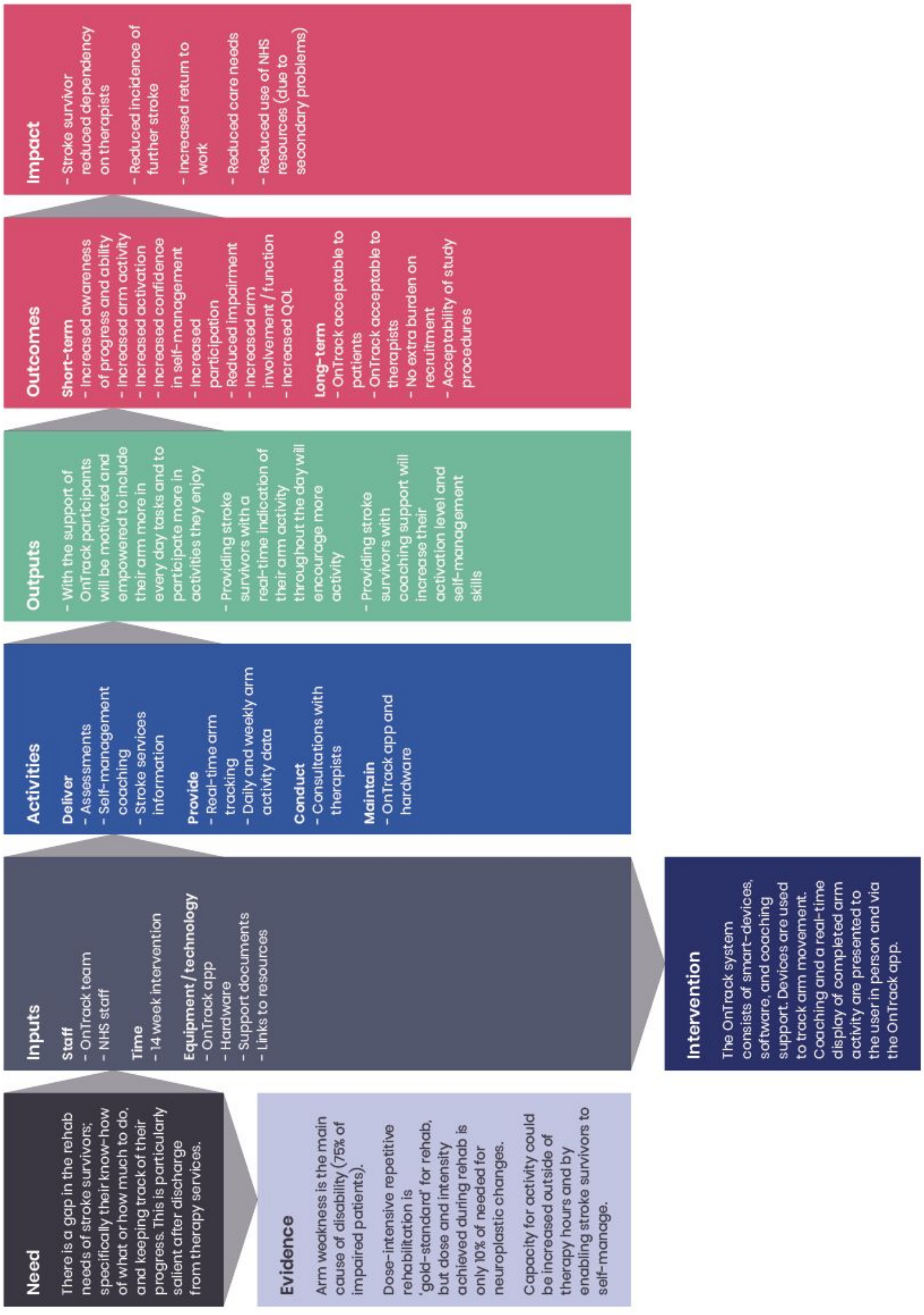

Figure 3

Examples of visualisations created using aggregated data captured by OnTrack from healthy beta testers between June-August 2019

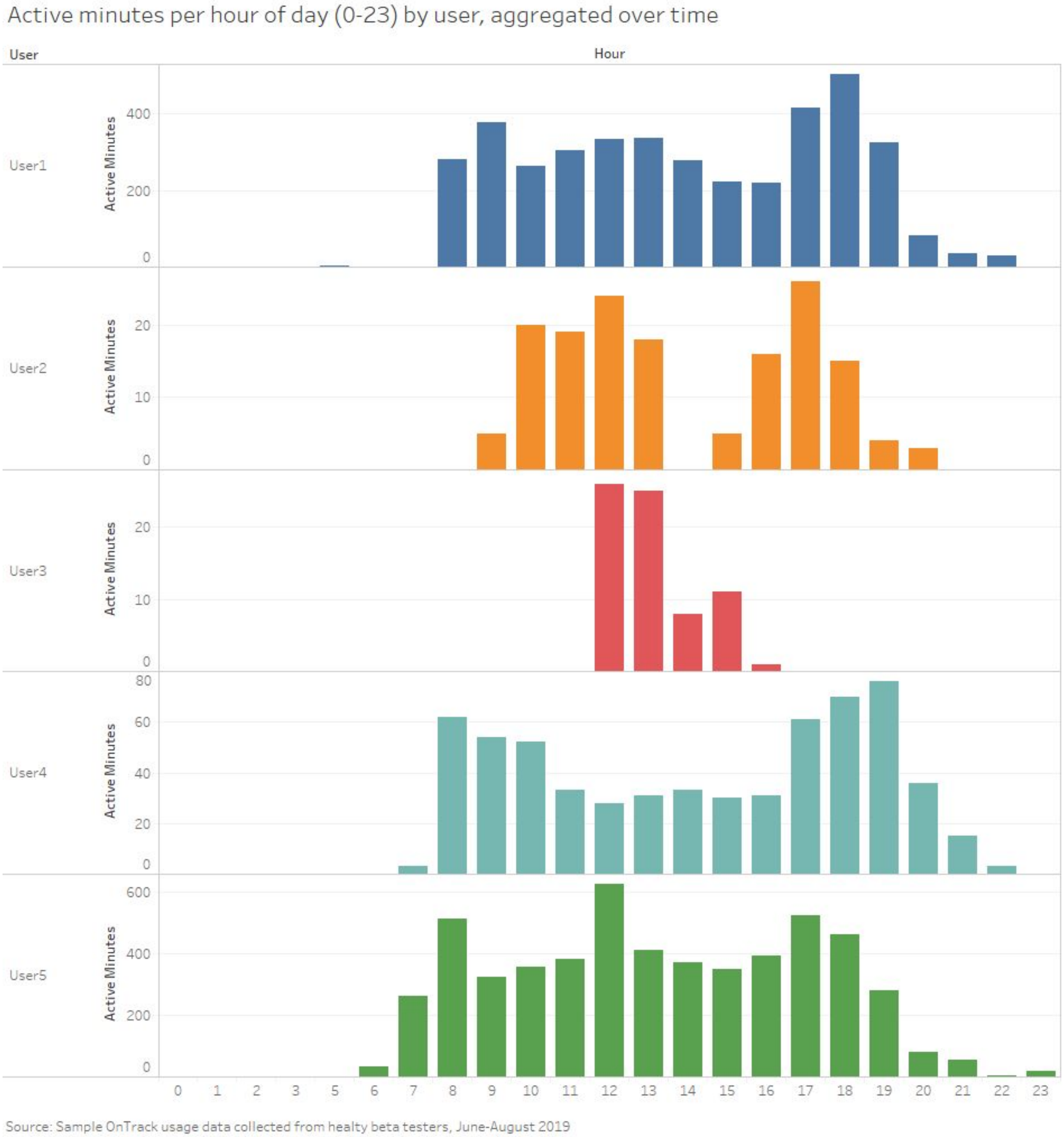

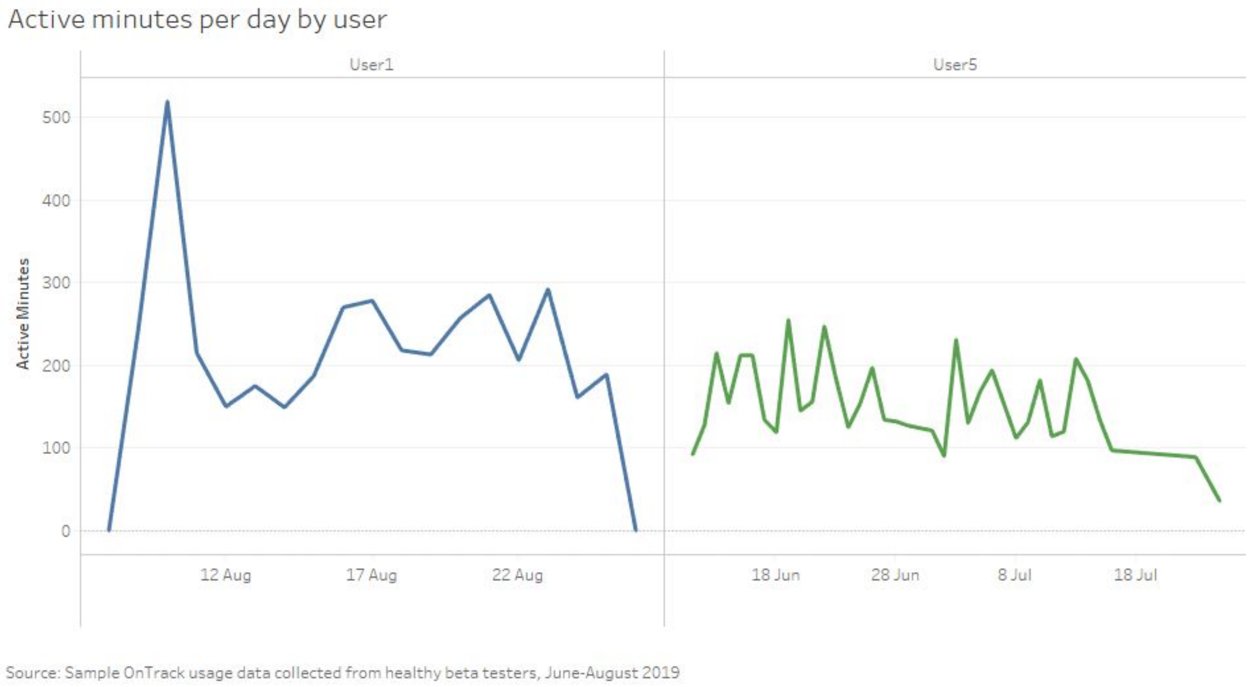
Active minutes per day by user
User1
User5
Active Minutes
500
400
300
200
100
0
12 Aug
17 Aug
22 Aug
18 Jun
28 Jun
8 Jul
18 Jul
Source: Sample OnTrack usage data collected from healthy beta testers, June-August 2019

Reporting checklist for protocol of a clinical trial.

Based on the SPIRIT guidelines.

Instructions to authors

Complete this checklist by entering the page numbers from your manuscript where readers will find each of the items listed below.

Your article may not currently address all the items on the checklist. Please modify your text to include the missing information. If you are certain that an item does not apply, please write "n/a" and provide a short explanation.

Upload your completed checklist as an extra file when you submit to a journal.

In your methods section, say that you used the SPIRITreporting guidelines, and cite them as:

Chan A-W, Tetzlaff JM, Altman DG, Laupacis A, Gøtzsche PC, Krleža-Jerić K, Hróbjartsson A, Mann H, Dickersin K, Berlin J, Doré C, Parulekar W, Summerskill W, Groves T, Schulz K, Sox H, Rockhold FW, Rennie D, Moher D. SPIRIT 2013 Statement: Defining standard protocol items for clinical trials. Ann Intern Med. 2013;158(3):200-207

For peer review only

		Reporting Item	Page Number
**Administrative information**			
Title	#1	Descriptive title identifying the study design, population, interventions, and, if applicable, trial acronym	1
Trial registration	#2a	Trial identifier and registry name. If not yet registered, name of intended registry	2
Trial registration: data set	#2b	All items from the World Health Organization Trial Registration Data Set	n/a
Protocol version	#3	Date and version identifier	11
Funding	#4	Sources and types of financial, material, and other support	12
Roles and responsibilities: contributorship	#5a	Names, affiliations, and roles of protocol contributors	11

Roles and responsibilities: sponsor contact information	#5b	Name and contact information for the trial sponsor	n/a
Roles and responsibilities: sponsor and funder	#5c	Role of study sponsor and funders, if any, in study design; collection, management, analysis, and interpretation of data; writing of the report; and the decision to submit the report for publication, including whether they will have ultimate authority over any of these activities	11
Roles and responsibilities: committees	#5d	Composition, roles, and responsibilities of the coordinating centre, steering committee, endpoint adjudication committee, data management team, and other individuals or groups overseeing the trial, if applicable (see Item 21a for data monitoring committee)	n/a
**Introduction**			
Background and rationale	#6a	Description of research question and justification for undertaking the trial, including summary of relevant studies (published and unpublished) examining benefits and harms for each intervention	3
Background and rationale: choice of comparators	#6b	Explanation for choice of comparators	n/a
Objectives	#7	Specific objectives or hypotheses	4
Trial design	#8	Description of trial design including type of trial (eg, parallel group, crossover, factorial, single group), allocation ratio, and framework (eg, superiority, equivalence, non-inferiority, exploratory)	5
**Methods: Participants, interventions, and outcomes**			
Study setting	#9	Description of study settings (eg, community clinic, academic hospital) and list of countries where data will be collected. Reference to where list of study sites can be obtained	5
Eligibility criteria	#10	Inclusion and exclusion criteria for participants. If applicable, eligibility criteria for study centres and individuals who will	5

For peer review only

		perform the interventions (eg, surgeons, psychotherapists)	
Interventions: description	#11a	Interventions for each group with sufficient detail to allow replication, including how and when they will be administered	6
Interventions: modifications	#11b	Criteria for discontinuing or modifying allocated interventions for a given trial participant (eg, drug dose change in response to harms, participant request, or improving / worsening disease)	n/a
Interventions: adherance	#11c	Strategies to improve adherence to intervention protocols, and any procedures for monitoring adherence (eg, drug tablet return; laboratory tests)	n/a
Interventions: concomitant care	#11d	Relevant concomitant care and interventions that are permitted or prohibited during the trial	n/a
Outcomes	#12	Primary, secondary, and other outcomes, including the specific measurement variable (eg, systolic blood pressure), analysis metric (eg, change from baseline, final value, time to event), method of aggregation (eg, median, proportion), and time point for each outcome. Explanation of the clinical relevance of chosen efficacy and harm outcomes is strongly recommended	7
Participant timeline	#13	Time schedule of enrolment, interventions (including any run-ins and washouts), assessments, and visits for participants. A schematic diagram is highly recommended (see Figure)	Table 2
Sample size	#14	Estimated number of participants needed to achieve study objectives and how it was determined, including clinical and statistical assumptions supporting any sample size calculations	6
Recruitment	#15	Strategies for achieving adequate participant enrolment to reach target sample size	6
**Methods: Assignment of interventions (for controlled trials)**			
Allocation: sequence generation	#16a	Method of generating the allocation sequence (eg, computer-generated random numbers), and list of any factors for stratification. To reduce predictability of a random sequence, details of any planned restriction (eg, blocking) should be provided in a separate document that is unavailable to those who enrol participants or assign interventions	n/a

For peer review only

Allocation concealment mechanism	#16b	Mechanism of implementing the allocation sequence (eg, central telephone; sequentially numbered, opaque, sealed envelopes), describing any steps to conceal the sequence until interventions are assigned	n/a
Allocation: implementation	#16c	Who will generate the allocation sequence, who will enrol participants, and who will assign participants to interventions	n/a
Blinding (masking)	#17a	Who will be blinded after assignment to interventions (eg, trial participants, care providers, outcome assessors, data analysts), and how	n/a
Blinding (masking): emergency unblinding	#17b	If blinded, circumstances under which unblinding is permissible, and procedure for revealing a participant's allocated intervention during the trial	n/a
**Methods: Data collection, management, and analysis**			
Data collection plan	#18a	Plans for assessment and collection of outcome, baseline, and other trial data, including any related processes to promote data quality (eg, duplicate measurements, training of assessors) and a description of study instruments (eg, questionnaires, laboratory tests) along with their reliability and validity, if known. Reference to where data collection forms can be found, if not in the protocol	9
Data collection plan: retention	#18b	Plans to promote participant retention and complete follow-up, including list of any outcome data to be collected for participants who discontinue or deviate from intervention protocols	n/a
Data management	#19	Plans for data entry, coding, security, and storage, including any related processes to promote data quality (eg, double data entry; range checks for data values). Reference to where details of data management procedures can be found, if not in the protocol	9
Statistics: outcomes	#20a	Statistical methods for analysing primary and secondary outcomes. Reference to where other details of the statistical analysis plan can be found, if not in the protocol	9
Statistics: additional analyses	#20b	Methods for any additional analyses (eg, subgroup and adjusted analyses)	9

For peer review only

Statistics: analysis population and missing data	#20c	Definition of analysis population relating to protocol non-adherence (eg, as randomised analysis), and any statistical methods to handle missing data (eg, multiple imputation)	n/a
**Methods: Monitoring**			
Data monitoring: formal committee	#21a	Composition of data monitoring committee (DMC); summary of its role and reporting structure; statement of whether it is independent from the sponsor and competing interests; and reference to where further details about its charter can be found, if not in the protocol. Alternatively, an explanation of why a DMC is not needed	n/a
Data monitoring: interim analysis	#21b	Description of any interim analyses and stopping guidelines, including who will have access to these interim results and make the final decision to terminate the trial	n/a
Harms	#22	Plans for collecting, assessing, reporting, and managing solicited and spontaneously reported adverse events and other unintended effects of trial interventions or trial conduct	n/a
Auditing	#23	Frequency and procedures for auditing trial conduct, if any, and whether the process will be independent from investigators and the sponsor	n/a
**Ethics and dissemination**			
Research ethics approval	#24	Plans for seeking research ethics committee / institutional review board (REC / IRB) approval	10
Protocol amendments	#25	Plans for communicating important protocol modifications (eg, changes to eligibility criteria, outcomes, analyses) to relevant parties (eg, investigators, REC / IRBs, trial participants, trial registries, journals, regulators)	10
Consent or assent	#26a	Who will obtain informed consent or assent from potential trial participants or authorised surrogates, and how (see Item 32)	6
Consent or assent: ancillary studies	#26b	Additional consent provisions for collection and use of participant data and biological specimens in ancillary studies, if applicable	n/a
Confidentiality	#27	How personal information about potential and enrolled participants will be collected, shared, and maintained in order to protect confidentiality before, during, and after the trial	10

For peer review only

Declaration of interests	#28	Financial and other competing interests for principal investigators for the overall trial and each study site	12
Data access	#29	Statement of who will have access to the final trial dataset, and disclosure of contractual agreements that limit such access for investigators	10
Ancillary and post trial care	#30	Provisions, if any, for ancillary and post-trial care, and for compensation to those who suffer harm from trial participation	n/a
Dissemination policy: trial results	#31a	Plans for investigators and sponsor to communicate trial results to participants, healthcare professionals, the public, and other relevant groups (eg, via publication, reporting in results databases, or other data sharing arrangements), including any publication restrictions	10
Dissemination policy: authorship	#31b	Authorship eligibility guidelines and any intended use of professional writers	n/a
Dissemination policy: reproducible research	#31c	Plans, if any, for granting public access to the full protocol, participant-level dataset, and statistical code	n/a
**Appendices**			
Informed consent materials	#32	Model consent form and other related documentation given to participants and authorised surrogates	n/a
Biological specimens	#33	Plans for collection, laboratory evaluation, and storage of biological specimens for genetic or molecular analysis in the current trial and for future use in ancillary studies, if applicable	n/a

The SPIRIT checklist is distributed under the terms of the Creative Commons Attribution License CC-BY-ND 3.0. This checklist was completed on 07. October 2019 using https://www.goodreports.org/, a tool made by the EQUATOR Network in collaboration with Penelope.ai

For peer review only

Protocol for a feasibility study of OnTrack: a digital system for upper-limb rehabilitation after stroke.

Journal:	*BMJ Open*
Manuscript ID	bmjopen-2019-034936.R1
Article Type:	Protocol
Date Submitted by the Author:	26-Dec-2019
Complete List of Authors:	Fusari, Gianpaolo; Royal College of Art, Helix Centre; Imperial College London, Helix Centre<br>Gibbs, Ella; Imperial College London, Helix Centre<br>Hoskin, Lily; Imperial College London, Helix Centre<br>Dickens, Daniel; Imperial College London, Helix Centre<br>Leis, Melanie; Imperial College London, Big Data and Analytical Unit, Institute of Global Health Innovation<br>Taylor, Elizabeth; Kingston and St George's Faculty of Health Social Care and Education<br>Jones, Fiona; Kingston and St George's Faculty of Health Social Care and Education<br>Darzi, Ara; Imperial College London, Helix Centre
**Primary Subject Heading**:	Neurology
Secondary Subject Heading:	Rehabilitation medicine, Neurology, Public health
Keywords:	Stroke < NEUROLOGY, REHABILITATION MEDICINE, NEUROLOGY, PUBLIC HEALTH

SCHOLARONE™
Manuscripts

I, the Submitting Author has the right to grant and does grant on behalf of all authors of the Work (as defined in the below author licence), an exclusive licence and/or a non-exclusive licence for contributions from authors who are: i) UK Crown employees; ii) where BMJ has agreed a CC-BY licence shall apply, and/or iii) in accordance with the terms applicable for US Federal Government officers or employees acting as part of their official duties; on a worldwide, perpetual, irrevocable, royalty-free basis to BMJ Publishing Group Ltd ("BMJ") its licensees and where the relevant Journal is co-owned by BMJ to the co-owners of the Journal, to publish the Work in this journal and any other BMJ products and to exploit all rights, as set out in our licence.

The Submitting Author accepts and understands that any supply made under these terms is made by BMJ to the Submitting Author unless you are acting as an employee on behalf of your employer or a postgraduate student of an affiliated institution which is paying any applicable article publishing charge ("APC") for Open Access articles. Where the Submitting Author wishes to make the Work available on an Open Access basis (and intends to pay the relevant APC), the terms of reuse of such Open Access shall be governed by a Creative Commons licence – details of these licences and which Creative Commons licence will apply to this Work are set out in our licence referred to above.

Other than as permitted in any relevant BMJ Author's Self Archiving Policies, I confirm this Work has not been accepted for publication elsewhere, is not being considered for publication elsewhere and does not duplicate material already published. I confirm all authors consent to publication of this Work and authorise the granting of this licence.

For peer review only

Title

Protocol for a feasibility study of OnTrack: a digital system for upper-limb rehabilitation after stroke.

Authors and affiliations

Gianpaolo Fusari[1], Ella Gibbs[1], Lily Hoskin[1], Daniel Dickens[1], Melanie Leis[2], Elizabeth Taylor[3], Fiona Jones[3], Ara Darzi[1]

[1]Helix Centre, Imperial College London and the Royal College of Art, London, United Kingdom
[2]Big Data and Analytical Unit, Institute of Global Health Innovation, Imperial College London, London, United Kingdom
[3]Kingston University and St George's, University of London, London, United Kingdom

Correspondence to:
Gianpaolo Fusari
HELIX Centre, Institute of Global Health Innovation
Imperial College London, 3rd Floor, Paterson Building, St Mary's Hospital, 16 South Wharf Road, London W2 1PF, UK
gianpaolo@helixcentre.com
+44 (0)77 6779 2770

Key words

Stroke, rehabilitation medicine, neurology, public health, digital health

Word count

4223

For peer review only

Abstract

Introduction

Arm weakness is a common problem after stroke (affecting 450,000 people in the UK) leading to loss of independence. Repetitive activity is critical for recovery but research shows people struggle with knowing what or how much to do, and keeping track of progress. Working with more than 100 therapists (occupational therapists and physiotherapists) and patients with stroke, we co-developed the OnTrack intervention - consisting of software for smart-devices and coaching support - that has the potential to address this problem. This is a protocol to assess the feasibility of OnTrack for evaluation in a randomised control trial.

Methods and analysis

A mixed methods, single-arm study design will be used to evaluate the feasibility of OnTrack for hospital and community use. A minimum sample of 12 participants from a stroke unit will be involved in the study for 14 weeks. During week 1, 8 and 14 participants will complete assessments relating to their arm function, arm impairment, and activation. During weeks 2-13 participants will use OnTrack to track their arm movement in real time, receive motivational messages, and face-to-face sessions to address problems, gain feedback on activity, and receive self-management skills coaching. All equipment will be loaned to study participants. A parallel process evaluation will be conducted to assess the intervention's fidelity, dose and reach, using a mixed methods approach. A Public and Patient Involvement (PPI) group will oversee the study and help with interpretation and dissemination of qualitative and quantitative data findings.

Ethics and dissemination

Ethical approval granted by the NHS Health Research Authority, Health and Care Research Wales, and the London - Surrey Research Ethics Committee (ref.19/LO/0881). Trial results will be submitted for publication in peer review journals, presented at international conferences and disseminated amongst stroke communities. The results of this trial will inform development of a definitive trial.

Trial registration details

ClinicalTrials.gov (NCT03944486), pre-results.

For peer review only

Strengths and limitations

- This is a feasibility trial of a novel intervention which employs an integrated approach for tracking arm activity and coaching with the aim of increasing stroke survivors' confidence and ability to use their impaired arm in daily activities, increasing the opportunities for repetitive rehabilitation (repeating a movement or series of movements with a rehabilitative or functional aim).
- Patient and Public Involvement (PPI) with more than 100 stroke survivors, carers, and clinicians have contributed to our needs-finding phase, co-designed OnTrack and informed the feasibility study. A new PPI group will oversee the running of the study and help with interpretation of qualitative and quantitative data findings.
- An independent process evaluation will provide detailed information about implementation, context, and the mechanisms of impact of the intervention. Findings will help in the understanding of intervention fidelity and training needs required for a definitive trial.
- For pragmatic reasons the study uses a non-randomised designed carried out at a single site- this will limit understanding about randomisation and recruitment
- Participants will not be followed-up after intervention period; however participant views will be sought regarding appropriate follow-up times in a subsequent definitive trial.

Introduction

Every year around the world over 15 million people experience a stroke, leaving 5 million people with a permanent disability.(1) Stroke is the leading cause of disability in the UK; half of the nearly 1.2 million stroke survivors who live in the country have some form of disability, significantly contributing to the loss of independence and feeling of isolation that they experience. (2),(3) Furthermore, stroke is estimated to cost UK society £26 billion every year, with the vast majority of these costs borne by the informal care sector.(2)

Upper-limb (arm) weakness is the main cause of physical impairment affecting 75% of disabled stroke survivors; this equates to around 450,000 people in the UK.(2) Dose-intensive repetitive rehabilitation is widely accepted as the 'gold-standard' for regaining ability after stroke, however, NHS resources are often limited and unable to provide this.(4) A recent Cochrane review of over 500 trials failed to yield high-quality practice recommendations for interventions for the upper-limb.(5) Arm recovery after stroke is a national research priority.(6) There is a correlation between physical activity after stroke and the ability to perform activities of daily living (most of which involve the use of the arm) nonetheless, studies suggest that the actual time patients are active is minimal.(7,8) Many current approaches to solving this problem focus on improving the prescribed rehabilitation sessions, often employing gamification techniques.(9,10) Whilst this is important, there is untapped potential to increase repetitive rehabilitation by targeting the large proportion of the day where patients are going about their daily activities and can use their arm movement

For peer review only

(however small) to a greater extent. Capacity for activity could be increased further by using self-management methods as demonstrated by several different programmes in stroke and other long-term conditions.(11-14) This has informed the development of OnTrack which aims to increase opportunities for activity by improving individuals' self-management skills through tailored support and real-time activity feedback on their arm movement.

An ethnographic study conducted by the Helix Centre (funded by Innovate UK) confirmed what other studies have shown (7,8,15) that patients struggle to see and keep track of improvements, this impacts their motivation and leaves them dependent on therapists for feedback. Stroke survivors often report feeling unsupported after leaving hospital and not knowing how to best help themselves improve their arm function.(16-18) Feedback gathered from over 100 stroke survivors and clinicians was the basis for developing the OnTrack intervention.

A proof-of-concept test of OnTrack gathered data from a small group of patients (n=7) and confirmed that the intervention was safe and generally users could understand how and when to use it. Participants reported they were more aware of their impaired arm and had increased confidence in using it for new tasks. A 20% mean increase in minutes of activity on the impaired arm was observed. The work conducted to date is unpublished and has some limitations however it has shaped the intervention and suggests that OnTrack has the potential to be a scalable solution that requires minimal training and could be used in conjunction with NHS services to help increase the overall amount of activity performed with the impaired arm. This study will assess the feasibility of the OnTrack intervention and inform the design of a definitive randomised controlled trial (RCT) to evaluate its clinical effectiveness, and follows the Standard Protocol Items: Recommendations for Interventional Trials (SPIRIT) guidelines.(19)

Methods and analysis

Aims and objectives

The primary aim is to evaluate the feasibility of an RCT to test the effectiveness of the OnTrack intervention for upper limb rehabilitation after stroke.

The objectives are to:

- Assess the feasibility of recruitment from hyper-acute and acute stroke units, and rehabilitation wards to ascertain strategy and recruitment rates.
- Assess dropout rates by observing adherence and compliance with the intervention.
- Understand the acceptability and usability of the intervention by stroke survivors.
- Understand the acceptability of study procedures by healthcare professionals.
- Explore implementation fidelity, dose and reach of the OnTrack intervention.

For peer review only

The study will also collect clinical outcomes regarding arm function, impairment and activation to identify an appropriate primary outcome, and to estimate parameters for a sample size calculation for an RCT (Table 1).

Study design

A feasibility study with a nested process evaluation (Figure 1). The study is a single-site, non-randomised intervention trial. The design of the study was developed through a collaborative approach between the study researchers, a PPI steering group, front-line therapists, and the Research Design Service at the National Institute for Health Research.

An independent process evaluation will be conducted in parallel to learn about usage and engagement mechanisms of participants, therapists and other frontline staff, providing critical information for implementation fidelity and impact mechanisms necessary for scale-up.

Study setting

The study will be conducted at an inner city NHS hospital Trust in London. Recruited participants will be able to continue to receive the intervention at home if discharged from hospital prior to ending the intervention period (14 weeks).

Participants

The inclusion criteria encompasses:

- Adults (aged 18 or over).
- Stroke diagnosis less than 6 months previously (first or recurrent). Some participants will be recruited from an in-patient rehabilitation ward, hence the 6 month post-stroke limit.
- Arm impairment of any type or level (including weakness - including dense hemiplegia, neglect, and sensory deficits). This to enable better understanding of which impairment level groups would benefit *or not* from using the intervention, especially considering the impact it may have on people's motivation regardless of their level of impairment.
- Ability to provide informed consent.
- Reliability to communicate (verbally or nonverbally) and understand English.
- Ability to read a predefined short message.

Potential participants who at the time of recruitment present with any of the following will be excluded:

- Unstable medical condition.
- Self reported "severe" pain in the arm affected either at rest or during movement.
- Severe oedema in the arm affected by their stroke, judged by the consenting therapist.
- Known discharge plans to a hospital other than the site Trust or residential care in less than 7 weeks (a small proportion of patients staying at CNRU may be in hospital for up to 12 weeks).

For peer review only

Recruitment

Participants will be recruited from the Hyperacute Stroke Unit (HASU), Acute Stroke Unit (ASU), and Clinical Neurorehabilitation Unit (CNRU) at an inner city NHS hospital Trust in London.

Stroke therapists (occupational therapists, physiotherapists) will be responsible for screening and identifying suitable patients. They will introduce the study to potential participants and provide information documents. Potential participants will be given a minimum of 24 hours to consider the advantages and disadvantages of participating in the study and to formulate questions. Therapists will be able to answer questions or will liaise with the research team to provide an answer. Once all questions are answered and a potential participant is willing to participate, consent will be taken by the therapist. Only at this stage will patient information be shared with the research team. There may be situations where a therapist is only able to take verbal consent from a participant due to time or material constraints, in such cases the researchers will be able to take written consent from the participant upon first meeting them.

For peer review only

Sample size calculation

Guidelines advocate a sample size of 12-30 participants for feasibility studies.(20) Experienced clinical academics and clinicians at the trial site have advised to expect about 50% of eligible patients to agree to participation and a 50% completion rate. This has informed a recruitment plan to identify at least 60 potential participants in a period of 30 weeks to reach the minimum sample size.

Intervention

The intervention is the OnTrack system as a whole. The system consists of smart-devices (smartphone and smartwatch), software (OnTrack app), and coaching support. Smart-devices are used to track arm movement. Motivational messages and a real-time display of completed arm activity (in minutes) are presented to the user via the OnTrack app. Coaching support is provided through fortnightly consultations by the researchers. During consultations, participants will receive self-management training informed by the Bridges Self-Management (21) and TaCAS (22) self-management programs. Coaching sessions are themed around principles of self-management (see Table 2, OnTrack Consultation column).

Data gathered by the OnTrack system can be accessed by the researchers via a digital dashboard to inform consultations.

Participants will be loaned all equipment necessary for the trial and no previous experience with using smart-devices is required to participate. Technical support will be provided only in cases where the hardware and/or software fail to perform the required functions to deliver the intervention.

Table 2 provides a participation schedule and a summary of the intervention procedures.

Outcomes

Feasibility of trial design and procedures

- Recruitment strategy and rates (feasibility of recruitment from HASU, ASU, CNRU wards) - percentage of patients: screened; eligible; approached; consented; excluded after screening. Participants consented and recruited will be logged by the research team in DOCUMAS(23)
- Compliance and adherence to intervention - measure of minutes of activity per participant as recorded by the OnTrack app, engagement with OnTrack app as measured by system analytics (for example: compliance with starting tracking arm activity daily, number of times and times of the day a particular screen is visited, the number of messages read and replied to, etc.)
- Completion rates - percentage of participants who complete the 14-week intervention period
- Acceptability and reasons for decline/withdrawal - number of participants who withdraw or decline the intervention and reasons why. A record of reasons for withdrawal and declining will be kept by the researchers. Reasons will be categorised in order of most common; this information will help the research team understand the reasons why someone might drop out or decline to participate in the study.

Clinical assessments

As a secondary objective, clinical outcomes will be collected at different time points by a qualified member of the research team to identify an appropriate primary outcome, and to estimate parameters for a sample size calculation for an RCT (Table 1). The outcome measures and assessments are listed below.

Patient activation

Patient activation is a concept recognised by the NHS that describes the knowledge, skills and confidence a person has in managing their own health and health care.(24) This will be measured using the Patient Activation Measure (PAM)(25) which has been validated in stroke populations in the UK.(26) The PAM survey measures patients on a 0–100 scale and can categorise patients into one of four activation levels along an empirically derived continuum.(25) Activation levels will be used to allocate participants one of three different OnTrack coaching tiers. The tiers aim to make the different aspects of the coaching more relevant and meaningful for the individual participant and their stage of recovery and self-management.

Arm impairment

Arm impairment will be measured objectively using the Fugl-Meyer Assessment for upper extremity (FMA-UE).(27) The FMA-UE has been tested extensively, and is found to have excellent psychometric properties and is recommended as core measures to be used in every stroke recovery and rehabilitation trial.(28)

For peer review only

Arm function

Arm function will be assessed using the Upper-Extremity Motor Activity Log-14 (MAL).(29) The MAL is a scripted, structured interview developed to self-report the amount and quality of use of the impaired arm in individuals with stroke in 14 different activities of daily living.

Gross level of disability

The modified Rankin Scale (mRS)(30) is the most prevalent functional outcome measure in contemporary stroke trials. The mRS quantifies disability using an ordinal hierarchical grading from zero (no symptoms) to 5 (severe disability).

Arm pain

Pain will be assessed using a visual analogue scale (VAS) from 0 (no pain) to 10 (excruciating pain) over the last 24 hours. VAS is a valid measure of pain intensity and is responsive to change.(31) Individuals scoring 3/10 or more in the affected arm will be withdrawn from the study unless their pain is only on movements that are not part of their usual everyday activities (e.g. arm pain when doing overhead reaching).

Cognitive impairment

Cognitive impairment will be assessed using the Montreal Cognitive Assessment (MoCA). The MoCA is a brief cognitive screening tool with high sensitivity and specificity for detecting mild cognitive impairment.(32) The MoCA defines impairment as follows: score of 18-25 = mild, 10-17 = moderate, <10 = severe.(32) Participants' scores will be used to look for associations between the use of OnTrack and any cognitive impairment.

Perceptual neglect

Albert's Test (AT) is being used to assess for unilateral spatial neglect (USN). This a simple test where participants are asked to cross out lines ruled in a standard fashion on a sheet of paper. If any lines are left uncrossed, and more than 70% of uncrossed lines are on the same side as motor deficit, USN is indicated. This may be quantified in terms of the percentage of lines left uncrossed. The test is very easy to administer and is a good predictor of functional activity six months after stroke onset.(33)

Quality of life

The EQ-5D-5L is a widely used standardised preference based measure of health status developed by the EuroQol Group in order to provide a simple, generic measure of health for clinical and economic appraisal.(34)

Additional assessments

A Lap-to-Table (LTT) timed test will be performed where the researchers measure the time it takes a participant to move their hand three times from resting on their lap to a table

For peer review only

positioned in front of them. This test is performed to assess its potential to use as part of the inclusion criteria for an RCT.

The NHS Friends and Family Test (FFT)(35) will be used to obtain feedback on the overall experience of using OnTrack and participating in the trial. Participants will be asked: “How likely are you to recommend OnTrack to friends and family if they needed similar care or treatment?” with answers provided in a Likert 5-point scale ranging from “extremely likely” to “extremely unlikely” and an “I don’t know” option.

The System Usability Scale (SUS) will be used to subjectively assess the usability of the OnTrack intervention. The test is a simple, ten-item scale covering a variety of aspects of system usability, such as the need for support, training, and complexity, and thus have a high level of face validity for measuring the usability of a system.(36)

Process evaluation

A process evaluation will be carried out by researchers working independently to the intervention team and in parallel to the trial to determine whether the OnTrack intervention was delivered as intended and to understand the mechanisms of impact. The aim of the process evaluation at the feasibility stage is mainly to understand how the trial design and intervention could be optimised ahead of an RCT.(37) A logic model(38,39) that defines the intervention in terms of inputs, outputs, causal assumptions and expected outcomes has been developed to help identify core questions for the evaluation team to explore (Figure 2). The evaluation team will observe 10% of all intervention sessions with the objective of documenting fidelity, dose and reach of the intervention.

Critical reflection and the process evaluation will help refine the intervention, as shown by mid-range theories (i.e. theories that help understand implementation).(40) Interim results will be shared with the intervention team at the half-way point with the objective to review some of the procedures and make minor adjustments as necessary.

In-depth semi-structured interviews will be conducted with patients at the end of their participation, a minimum sample of 12 is anticipated. A topic guide with themes drawing from the logic model will be used. Interviews will focus on participants’ experiences using OnTrack, their perceptions of arm tracking, motivational messaging and the researcher consultations. Additionally, the interviews will explore participants’ perceptions of the impact OnTrack had on them in terms of progress, awareness, participation, and confidence in self-management. Participants’ responses will be compared against activity data collected from the OnTrack app.

NHS therapists caring for participants taking part will be consented and invited to complete a short online survey to gather their feedback regarding acceptability of study procedures, they have the option to respond anonymously. The total number of therapists involved is difficult to predict as there may be team changes and staff movement during the course of the study. The survey will ask questions around three themes: 1) participation, relevance, quality and time spent in study procedures; 2) opinions on the benefit/detriment OnTrack

For peer review only

may have for patients; 3) opinions on how the intervention may or may not fit with service provision and their workflow.

Data analysis

Analysis will be completed on the parameters and implementation of the study in addition to the usability of OnTrack.

Data collected for the process evaluation will be a combination of qualitative data from interviews with stroke participants and therapists to explore their experiences of using OnTrack, as well as quantitative data on usage of OnTrack and the self-reported SUS. OnTrack therapy support sessions will be monitored through a fidelity checklist and observations (10 live sessions will be observed in total. In addition, the evaluation team will have access to recorded sessions that can be observed at their discretion). Interview data will undergo thematic analysis by the evaluation team. Data will be entered into NVIVO,(41) line by line coding and analysis will be informed by Braun and Clark's approach to thematic analysis.(42)

Changes over time will be evaluated in both OnTrack usage and outcome measures.

For OnTrack usage, the team will analyse users' activity patterns by day and hour of day. Figure 3 illustrates examples of visualisations created using aggregated data captured by OnTrack from healthy beta testers between June-August 2019. It compares users on active minutes per hour of day (aggregated over time) and active minutes per day.

OnTrack also captures specific usage metrics, including:

- Number of times OnTrack messages were opened
- Number of times daily and weekly activity were viewed on the phone
- Number of swipes on watch to reveal activity graph

These values will be plotted against the users' minutes of activity to better understand the potential impact of the app on activity over time.

The self-reported PAM will be captured at weeks 1, 8 and 14 for each user. It will be analysed in relation to the minutes of activity of each user over time to better understand the potential impact of the app on their levels of activation. SUS will be captured at weeks 8 and 14 and will be compared against actual usage metrics (described above) to assess usability.

Subgroup analyses are planned based on patient demographics, stroke disability (measured by mRankin scale at start of participation), stroke subtype and the care pathway patients go through during the intervention period.

The number of subgroups that will be available for analyses will depend on the characteristics of the participants. Whilst it's clear that the sample size will be relatively small, it is valuable to understand how we might approach subgroup analysis in a definitive trial with a larger sample size.

For peer review only

All data will be stored and accessed in accordance with GDPR guidance.

Clinical trial support will be provided by the Big Data and Analytical Unit (BDAU) at Imperial College London's Institute of Global Health Innovation (IGHI).

Patient and public involvement (PPI)

To date, over 100 stroke survivors, carers and therapists have been involved in the design of OnTrack. Participants and have been instrumental in highlighting areas for improvement in upper-limb stroke rehabilitation. They have contributed to a co-design process (including workshops, interviews, observations and surveys) resulting in the design, development and initial testing of OnTrack.

A steering group comprising of four stroke survivors was formed for the purpose of this feasibility study. Diversity within the group - both in terms of demographics and stroke severity - was considered. The group has supervised the development of all patient-facing material ensuring its clarity. They will also participate in data analysis by helping to refine themes and key messages arising from qualitative interviews. Participants will be trained by experienced researchers for this purpose.

The steering group will meet five times over the duration of the study, including an initial briefing session at the start to outline their involvement. Steering group members will be key members of the research team and their time and travel will be reimbursed according to INVOLVE(43) guidelines.

The PPI involvement plan was shared with Imperial College London's PPI 'Research Partners Group' on 21.02.19 who felt that the needs of the steering group have been accounted for.

Ethics and dissemination

The OnTrack study will be conducted in accordance with the recommendations for physicians involved in research on human subjects adopted by the 18th World Medical Assembly, Helsinki 1964 and later revisions; and in compliance with the relevant UK and European legislation including the NHS Health Research Authority (HRA) policy frameworks and the General Data Protection Regulation 2018 (GDPR).

The study was granted ethical approval by the HRA, Health and Care Research Wales, and the London - Surrey Research Ethics Committee (ref. 19/LO/0881). Local site capacity and capability approval has been granted by the hospital Trust.

The current approved protocol version is V1.3 dated 19.06.2019. Protocol amendments will be submitted for approval to the NHS HRA in the first instance and to the local site thereafter ahead of implementation.

For peer review only

The Chief Investigator is responsible for preserving the confidentiality of participants taking part in the study. Researchers will have patients' names, contact numbers, emails and home addresses for the purposes of arranging visits. This information will be stored in accordance with GDPR legislation. Participants are free to withdraw from the study at any time. However, anonymised activity data collected may still be used for data analysis as this is unlinked of any patient identifiable information.

The day-to-day management of the study will be coordinated by the Helix Centre. A study steering committee formed by the intervention team, evaluation team, PPI group, and representatives from the local site will meet at regular intervals throughout the study.

Regular updates about the trial will be made available through social media, blog posts, newsletters and the Helix Centre website (www.helixcentre.com). Trial results will be submitted for publication in journals, presented at national and international stroke meetings and conferences and disseminated amongst stroke communities.

Trial status

The first participant was enrolled on 09.09.2019 and recruitment is expected to complete by the end of March 2020. Enrolment and data collection was continuing as planned at the time of submission of this protocol.

Author statement

AD is grant holder and has project oversight along with DD. GF, EG and LH developed the intervention and conceived of the study. GF, EG, and FJ initiated the study design and ET and ML helped with further refinement. EG and GF are responsible for delivering the intervention and data collection. FJ and ET are responsible for the process evaluation. ML provides statistical expertise in trial design and is conducting the primary statistical analysis. All authors contributed to the refinement of the study protocol and approved the final manuscript.

Funding

This study is funded by the NIHR Imperial Biomedical Research Centre (BRC), grant 1215-20013. The views expressed are those of the author(s) and not necessarily those of the NIHR or the Department of Health and Social Care.

All intellectual property associated with the OnTrack system is owned by the Helix Centre which is a collaboration between Imperial College London and the Royal College of Art.

Data statement

Anonymised data will be made available in a public repository once the data have obtained validation through publication.

For peer review only

Conflict of interests

FJ is the founder of the social enterprise Bridges Self-Management. She has not received any financial support for this work that could have influenced the design.

Acknowledgments

The authors would like to acknowledge the contributions of the members of the PPI group; Jennifer Crow and Sarah Daniels for their input on recruitment and sample size calculations; and Dr Gaby Judah for her help in defining some aspects of the intervention.

Figure legends

Figure 1

Trial diagram

Figure 2

Logic model

Figure 3

Examples of visualisations created using aggregated data captured by OnTrack from healthy beta testers. Data for a minimum of 5 and a maximum of 18 days were aggregated for the period between June and August 2019.

For peer review only

Table 1

Outcome measures

Concept	Assessment	Week of administration
Patient Activation / Engagement	Patient Activation Measure (PAM)	1, 8, 14
Arm impairment	Fugl-Meyer Assessment for upper extremity (FMA-UE)	1, 8, 14
Arm function	Upper-Extremity Motor Activity Log-14 (MAL)	1, 8, 14
Gross level of disability	modified Rankin Scale (mRS)	1, 8, 14
Arm pain	Visual Analogue Scale (VAS)	1, 8, 14
Cognitive impairment	Montreal Cognitive Assessment (MoCA)	1, 8, 14
Arm neglect	Albert’s Test (AT)	1, 8, 14
Quality of life	EQ-5D-5L	1, 8, 14
Arm function	Lap-to-Table (LTT)	1, 8, 14
Service experience	Friends and Family Test (FTT)	8, 14
System usability	System Usability Scale (SUS)	14

For peer review only

Table 2

Intervention and Participation schedule

Week	Phase	Description	OnTrack consultation	Assessments
0	Information and consent	NHS therapists screen for eligible patients, provide information and consent participants		Screening, information, and consent
1	Baseline assessment (initial)	Participants complete outcome measures and wear activity trackers (Axivity AX3) on both arms during waking hours (typically 12 hours per day) for one week to gather accelerometer data which is translated into minutes of activity. This data creates a baseline of activity allowing left-to-right arm usage comparison		PAM, FMA-UE, MAL, mRS, VAS, MoCA, AT, EQ-5D-5L, LTT
2	OnTrack intervention	Participants wear a smartwatch (Apple Watch Series 3 or 4) on their impaired arm only during waking hours (typically 12 hours per day). They will receive real-time feedback on the amount of movement completed (measured in minutes) and daily motivational messages. Participants will receive fortnightly consultations with a researcher to troubleshoot and receive self-management skills training<br><br>Baseline assessments are repeated during week 8 (halfway)	Onboarding	
3			Check-in & self-management skills training (Problem Solving)	
4				
5			Check-in & self-management skills training (Self-Discovery)	
6				
7			Check-in & self-management skills training (Goal Setting)	
8			Halfway assessment<br><br>Check-in & self-management skills training (Goal Setting	PAM, FMA-UE, MAL, mRS, VAS, MoCA, AT, EQ-5D-5L, LTT, FFT

For peer review only

			cont.)	
9				
10			Check-in & self-management skills training (Reflection)	
11				
12			Check-in & self-management skills training (Sign-posting)	
13				
14	Baseline assessment (exit)	Participants complete outcome measures and wear activity trackers (Axivity AX3) on both arms during waking hours (typically 12 hours per day) for one week to gather accelerometer data which is translated into minutes of activity. This data creates a baseline of activity allowing left-to-right arm usage comparison		PAM, FMA-UE, MAL, mRS, VAS, MoCA, AT, EQ-5D-5L, LTT, FFT, SUS
15	Feedback	Independent evaluator leads feedback sessions with participants who have completed the intervention<br><br>End of participation		Semi-structured interview, online survey (therapists)

References

References
(1) WHO. *The Atlas of Heart Disease and Stroke.* Available from: https://www.who.int/cardiovascular_diseases/resources/atlas/en/ [Accessed 04 Oct 2019].
(2) Stroke Association. *State of the Nation: stroke statistics.* Stroke Association. 2018.
(3) Salter K, Hellings C, Foley N, Teasell R. The Experience of Living with Stroke: A Qualitative Meta-Synthesis. *Journal of Rehabilitation Medicine.* 2008; 40 (8): 595-602. Available from: doi: 10.2340/16501977-0238 Available from: http://www.ingentaconnect.com/content/mjl/sreh/2008/00000040/00000008/art00003 .

(4) Care Quality Commission. *Supporting life after stroke: a review of services for people who have had a stroke and their carers.* London: Care Quality Commission. 2011.
(5) Pollock A, Farmer SE, Brady MC, Langhorne P, Mead GE, Mehrholz J, et al. Interventions for improving upper limb function after stroke. *The Cochrane database of systematic reviews.* 2014; 2014 (11): CD010820. Available from: doi: 10.1002/14651858.CD010820.pub2 Available from: https://www.ncbi.nlm.nih.gov/pubmed/25387001 .
(6) Pollock A, St George B, Fenton M, Firkins L. Top 10 Research Priorities Relating to Life after Stroke – Consensus from Stroke Survivors, Caregivers, and Health Professionals. *International Journal of Stroke.* 2014; 9 (3): 313-320. Available from: doi: 10.1111/j.1747-4949.2012.00942.x Available from: https://journals.sagepub.com/doi/full/10.1111/j.1747-4949.2012.00942.x .
(7) Kunkel D, Fitton C, Burnett M, Ashburn A. Physical inactivity post-stroke: a 3-year longitudinal study. *Disability & Rehabilitation.* 2015; 37 (4): 304-310. Available from: doi: 10.3109/09638288.2014.918190 Available from: http://www.tandfonline.com/doi/abs/10.3109/09638288.2014.918190 .
(8) Bernhardt J, Dewey H, Thrift A, Donnan G. Inactive and Alone: Physical Activity Within the First 14 Days of Acute Stroke Unit Care. *Stroke.* 2004; 35 (4): 1005-1009. Available from: doi: 10.1161/01.STR.0000120727.40792.40 Available from: http://stroke.ahajournals.org/cgi/content/abstract/35/4/1005 .
(9) Laver KE, Lange B, George S, Deutsch JE, Saposnik G, Crotty M. Virtual reality for stroke rehabilitation. *The Cochrane database of systematic reviews.* 2017; 11 (11): CD008349. Available from: doi: 10.1002/14651858.CD008349.pub4 Available from: https://www.ncbi.nlm.nih.gov/pubmed/29156493 .
(10) Loureiro R, Harwin W, Nagai K, Johnson M. Advances in upper limb stroke rehabilitation: a technology push. *Medical & Biological Engineering & Computing.* 2011; 49 (10): 1103-1118. Available from: doi: 10.1007/s11517-011-0797-0 Available from: https://www.ncbi.nlm.nih.gov/pubmed/21773806 .
(11) Jones F, Gage H, Drummond A, Bhalla A, Grant R, Lennon S, et al. Feasibility study of an integrated stroke self-management programme: a cluster-randomised controlled trial. *BMJ Open.* 2016; 6 (1): e008900. Available from: doi: 10.1136/bmjopen-2015-008900 Available from: http://dx.doi.org/10.1136/bmjopen-2015-008900 .
(12) Harwood M, Weatherall M, Talemaitoga A, Barber PA, Gommans J, Taylor W, et al. Taking charge after stroke: promoting self-directed rehabilitation to improve quality of life – a randomized controlled trial. *Clinical Rehabilitation.* 2012; 26 (6): 493-501. Available from: doi: 10.1177/0269215511426017 Available from: https://journals.sagepub.com/doi/full/10.1177/0269215511426017 .
(13) Kendall E, Catalano T, Kuipers P, Posner N, Buys N, Charker J. Recovery following stroke: The role of self-management education. *Social Science & Medicine.* 2007; 64 (3): 735-746. Available from: doi: 10.1016/j.socscimed.2006.09.012 Available from: https://www.sciencedirect.com/science/article/pii/S0277953606004801 .
(14) Grady PA, Gough LL. Self-Management: A Comprehensive Approach to Management of Chronic Conditions. *American journal of public health.* 2014; 104 (8): e25-e31. Available from: doi: 10.2105/AJPH.2014.302041 Available from: https://www.ncbi.nlm.nih.gov/pubmed/24922170 .
(15) Esmonde T, McGinley J, Wittwer J, Goldie P, Martin C. Stroke rehabilitation: patient activity during non-therapy time. *Australian Journal of Physiotherapy.* 1997; 43 (1): 43-51. Available from: doi: 10.1016/S0004-9514(14)60401-3 Available from: https://www.sciencedirect.com/science/article/pii/S0004951414604013 .
(16) Buckland D. Leaving hospital is 'like falling off a cliff' for stroke survivors. *Raconteur.* APRIL 27, 2017 . Available from: https://www.raconteur.net/healthcare/leaving-hospital-is-like-falling-off-a-cliff-for-stroke-survivors [Accessed Oct 04 2019].
(17) McKevitt C, Fudge N, Redfern J, Sheldenkar A, Crichton S, Rudd AR, et al. Self-Reported

For peer review only

Long-Term Needs After Stroke. *Stroke.* 2011; 42 (5): 1398-1403. Available from: doi: 10.1161/STROKEAHA.110.598839 Available from: https://www.ncbi.nlm.nih.gov/pubmed/21441153 .
(18) Woodman P, Riazi A, Pereira C, Jones F. Social participation post stroke: a meta-ethnographic review of the experiences and views of community-dwelling stroke survivors. *Disability & Rehabilitation.* 2014; 36 (24): 2031-2043. Available from: doi: 10.3109/09638288.2014.887796 Available from: http://www.tandfonline.com/doi/abs/10.3109/09638288.2014.887796 .
(19) Chan A, Tetzlaff JM, Altman DG, Laupacis A, Gøtzsche PC, Krleža-Jerić K, et al. SPIRIT 2013 Statement: Defining Standard Protocol Items for Clinical Trials. *Annals of internal medicine.* 2013; 158 (3): 200-207. Available from: doi: 10.7326/0003-4819-158-3-201302050-00583 Available from: https://www.ncbi.nlm.nih.gov/pubmed/23295957 .
(20) Billingham SAM, Whitehead AL, Julious SA. An audit of sample sizes for pilot and feasibility trials being undertaken in the United Kingdom registered in the United Kingdom Clinical Research Network database. *BMC medical research methodology.* 2013; 13 (1): 104. Available from: doi: 10.1186/1471-2288-13-104 Available from: https://www.ncbi.nlm.nih.gov/pubmed/23961782 .
(21) Bridges Self-Management Limited. *Bridges Self-Management.* Available from: http://www.bridgesselfmanagement.org.uk/ [Accessed 07 Oct 2019].
(22) Medical Research Institute of New Zealand. *Taking Charge After Stroke (TaCAS).* Available from: https://mrinz.ac.nz/programmes/stroke/ [Accessed 07 oct 2019].
(23) DOCUMAS. *DOCUMAS: efficient health research management.* Available from: https://www.documas.eu/ [Accessed 04 Oct 2019].
(24) Hibbard J, Gilburt H. *Supporting people to manage their health An introduction to patient activation.* London: The Kings Fund. 2014.
(25) Hibbard JH, Stockard J, Mahoney ER, Tusler M. Development of the Patient Activation Measure (PAM): Conceptualizing and Measuring Activation in Patients and Consumers. *Health Services Research.* 2004; 39 (4p1): 1005-1026. Available from: doi: 10.1111/j.1475-6773.2004.00269.x Available from: https://onlinelibrary.wiley.com/doi/abs/10.1111/j.1475-6773.2004.00269.x .
(26) Roberts NJ, Kidd L, Dougall N, Patel IS, McNarry S, Nixon C. Measuring patient activation: The utility of the Patient Activation Measure within a UK context—Results from four exemplar studies and potential future applications. *Patient Education and Counseling.* 2016; 99 (10): 1739-1746. Available from: doi: 10.1016/j.pec.2016.05.006 Available from: https://www.clinicalkey.es/playcontent/1-s2.0-S0738399116301938 .
(27) Sahlgrenska Academy Institute of Neuroscience and Physiology, University of Gothenburg. *Fugl-Meyer.* Available from: https://neurophys.gu.se/english/departments/clinical_neuroscience_and_rehabilitation/rehabilitation-medicine/fugl-meyer [Accessed 04 Oct 2019].
(28) Pamela W Duncan, Martha Propst, Steven G Nelson. Reliability of the Fugl-Meyer Assessment of Sensorimotor Recovery Following Cerebrovascular Accident. *Physical Therapy.* 1983; 63 (10): 1606-1610. Available from: doi: 10.1093/ptj/63.10.1606 Available from: http://ptjournal.apta.org/content/63/10/1606.abstract .
(29) Uswatte G, Taub E, Morris D, Vignolo M, McCulloch K. Reliability and Validity of the Upper-Extremity Motor Activity Log-14 for Measuring Real-World Arm Use. *Stroke.* 2005; 36 (11): 2493-2496. Available from: doi: 10.1161/01.STR.0000185928.90848.2e Available from: http://stroke.ahajournals.org/cgi/content/abstract/36/11/2493 .
(30) Quinn TJ, Dawson J, Walters MR, Lees KR. Exploring the Reliability of the Modified Rankin Scale. *Stroke.* 2009; 40 (3): 762-766. Available from: doi: 10.1161/STROKEAHA.108.522516 Available from: http://stroke.ahajournals.org/cgi/content/abstract/40/3/762 .
(31) Jensen MP, Karoly P, Braver S. The measurement of clinical pain intensity: a comparison

For peer review only

of six methods. *Pain.* 1986; 27 (1): 117-126. Available from: doi: 10.1016/0304-3959(86)90228-9 Available from: https://www.sciencedirect.com/science/article/pii/0304395986902289 .
(32) Nasreddine ZS, Phillips NA, Bédirian V, Charbonneau S, Whitehead V, Collin I, et al. The Montreal Cognitive Assessment, MoCA: A Brief Screening Tool For Mild Cognitive Impairment. *Journal of the American Geriatrics Society.* 2005; 53 (4): 695-699. Available from: doi: 10.1111/j.1532-5415.2005.53221.x Available from: https://onlinelibrary.wiley.com/doi/abs/10.1111/j.1532-5415.2005.53221.x .
(33) Fullerton KJ, McSherry D, Stout RW. Albert's test: a neglected test of perceptual neglect. *Lancet (London, England).* 1986; 1 (8478): 430. Available from: https://www.ncbi.nlm.nih.gov/pubmed/2868349 .
(34) EuroQol - a new facility for the measurement of health-related quality of life. *Health policy.* 1990; 16 (3): 199-208. Available from: doi: 10.1016/0168-8510(90)90421-9 Available from: https://www.sciencedirect.com/science/article/pii/0168851090904219 .
(35) NHS England. *The Friends and Family Test.* NHS England. 2014.
(36) SUS: A 'Quick and Dirty' Usability Scale. *Usability Evaluation In Industry.* CRC Press; 1996. pp. 207-212.
(37) Moore GF, Audrey S, Barker M, Bond L, Bonell C, Hardeman W, et al. Process evaluation of complex interventions: Medical Research Council guidance. *BMJ : British Medical Journal.* 2015; 350 (mar19 6): h1258. Available from: doi: 10.1136/bmj.h1258 Available from: http://dx.doi.org/10.1136/bmj.h1258 .
(38) W.K. Kellogg Foundation. *Logic Model Development Guide.* Battle Creek, Michigan: W.K. Kellogg Foundation. 2004.
(39) The Scottish Government. *Designing and Evaluating Behaviour Change Interventions.* The Scottish Government. 2015.
(40) Kislov R, Pope C, Martin GP, Wilson PM. Harnessing the power of theorising in implementation science. *Implementation Science.* 2019; 14 (1): 103. Available from: doi: 10.1186/s13012-019-0957-4 Available from: https://search.proquest.com/docview/2324921962 .
(41) QSR International Ltd. *NVIVO.* Available from: https://www.qsrinternational.com/nvivo/nvivo-products [Accessed 07 Oct 2019].
(42) Braun V, Clarke V. Using thematic analysis in psychology. *Qualitative Research in Psychology.* 2006; 3 (2): 77-101. Available from: doi: 10.1191/1478088706qp063oa Available from: http://www.tandfonline.com/doi/abs/10.1191/1478088706qp063oa .
(43) National Institute for Health Research. *About INVOLVE.* Available from: https://www.invo.org.uk/about-involve/ [Accessed 04 Oct 2019].

For peer review only

Figure 1

Trial diagram

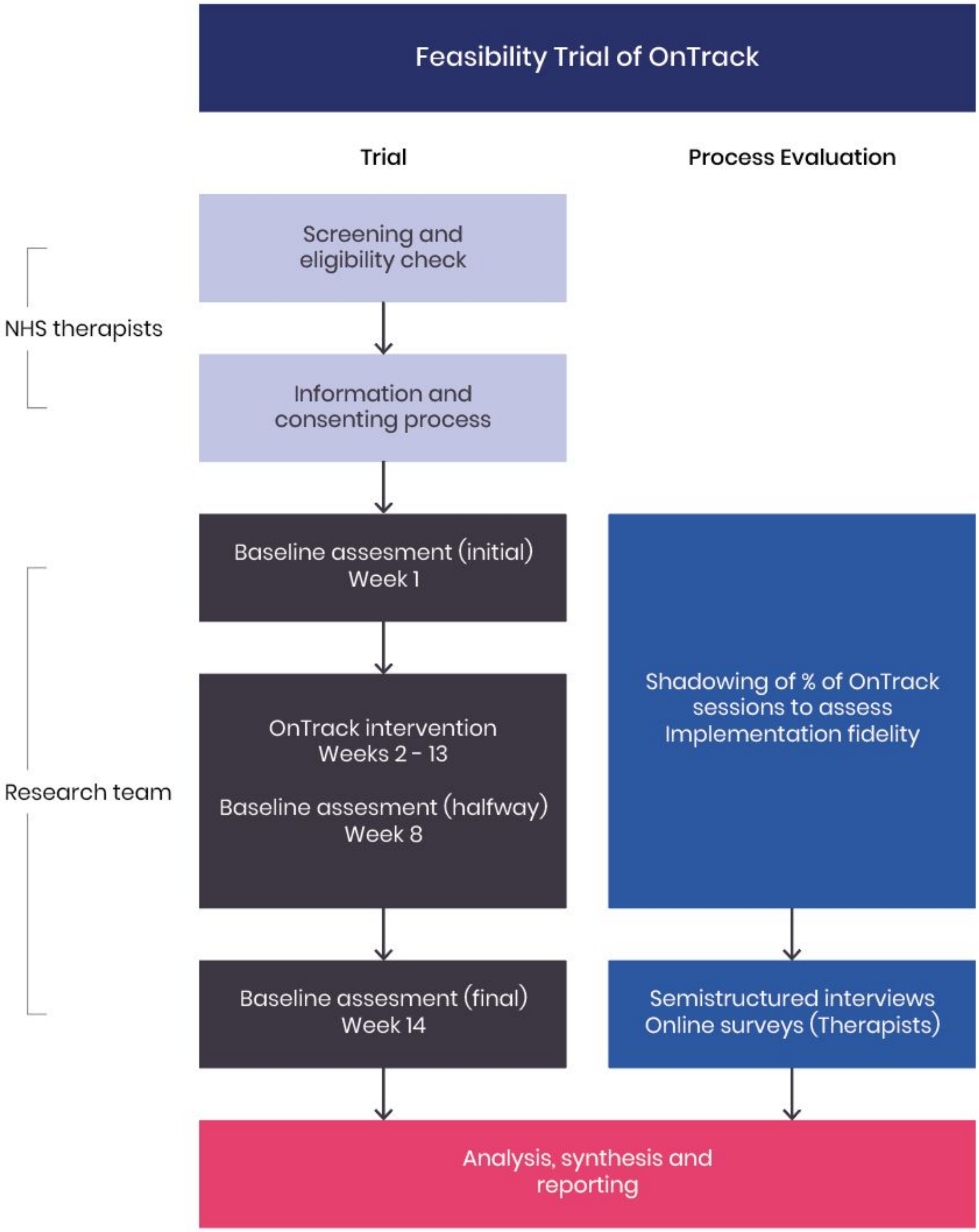

Figure 2

Logic model

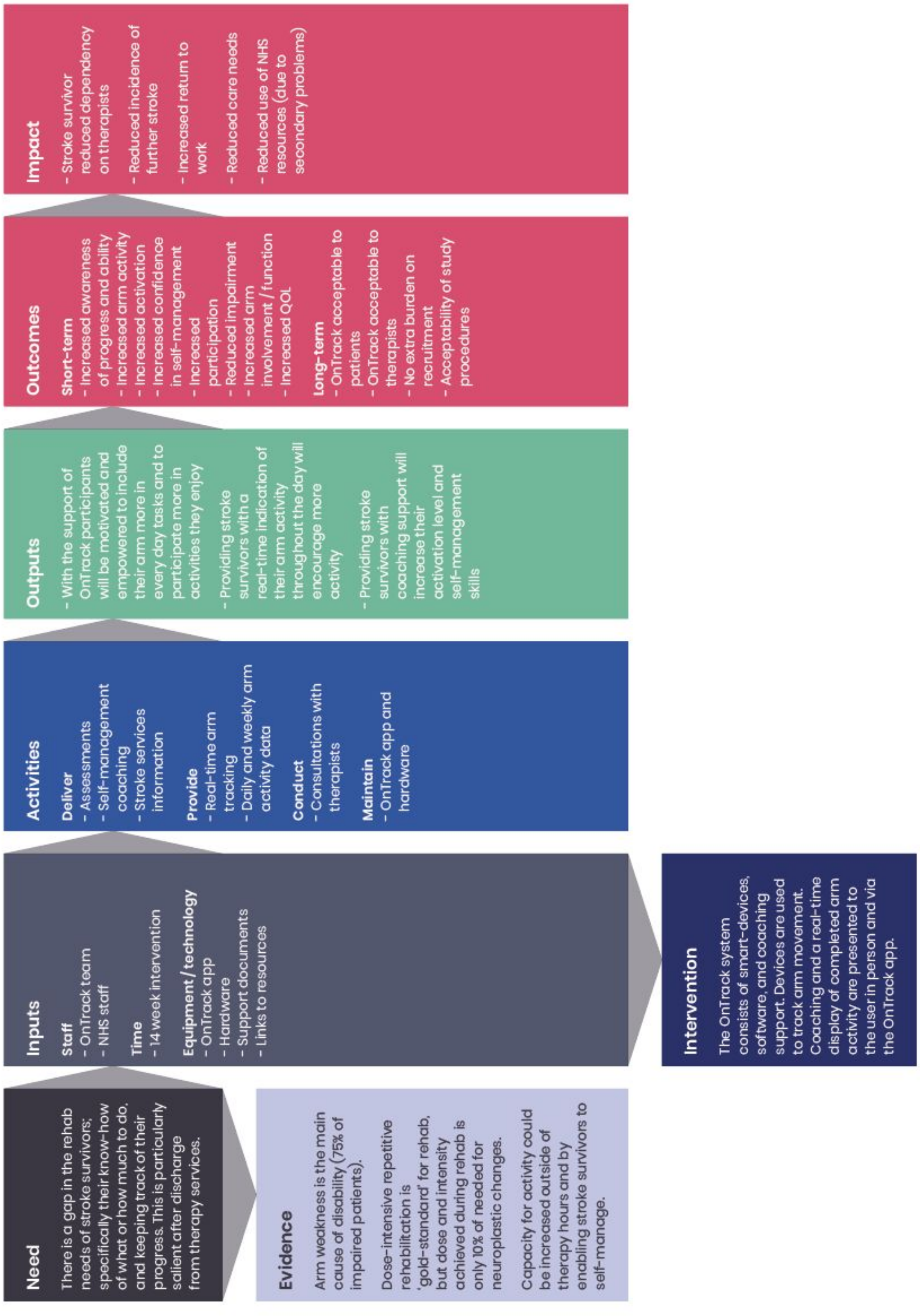

Figure 3

Examples of visualisations created using aggregated data captured by OnTrack from healthy beta testers between June-August 2019

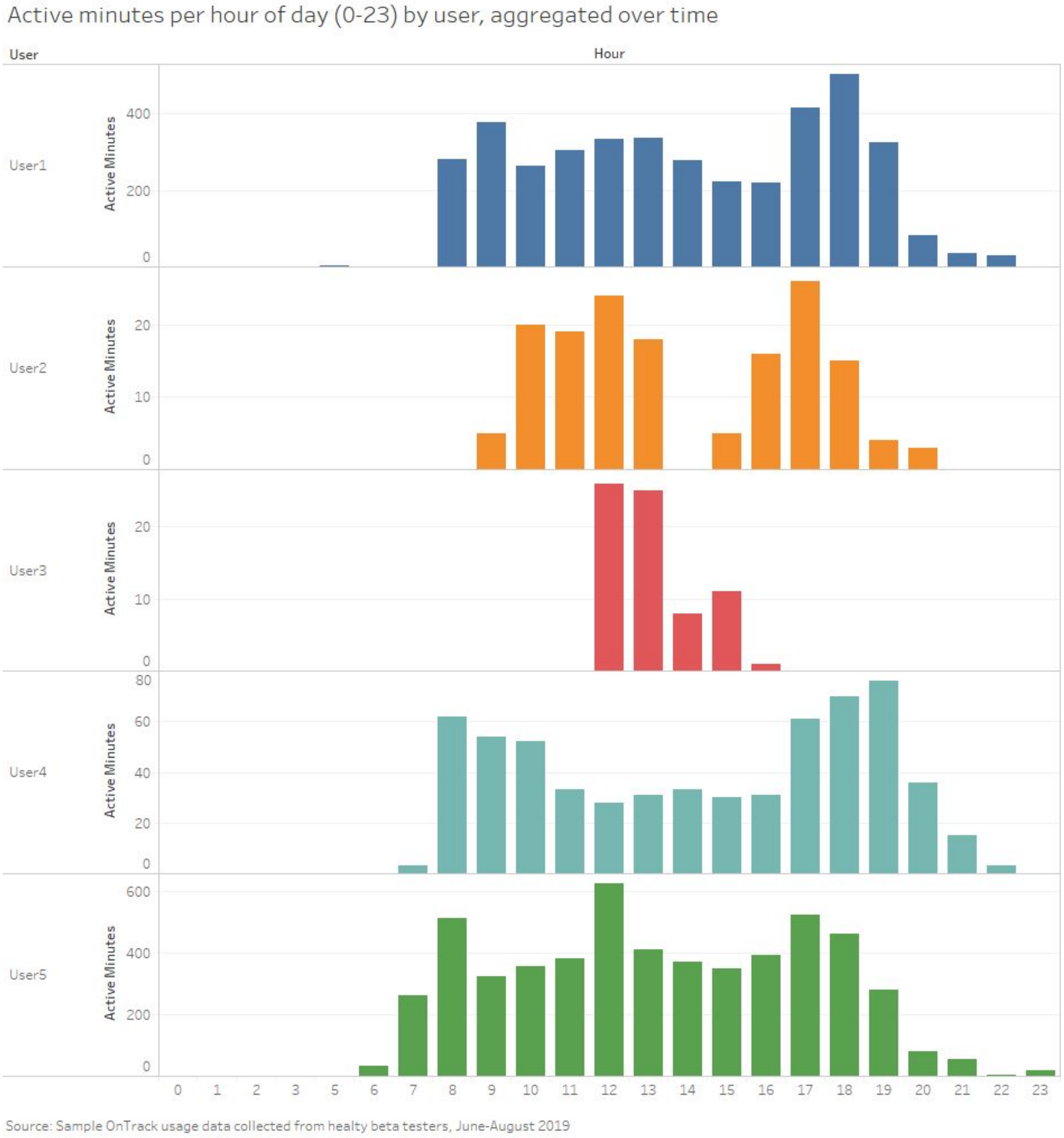

Active minutes per day by user

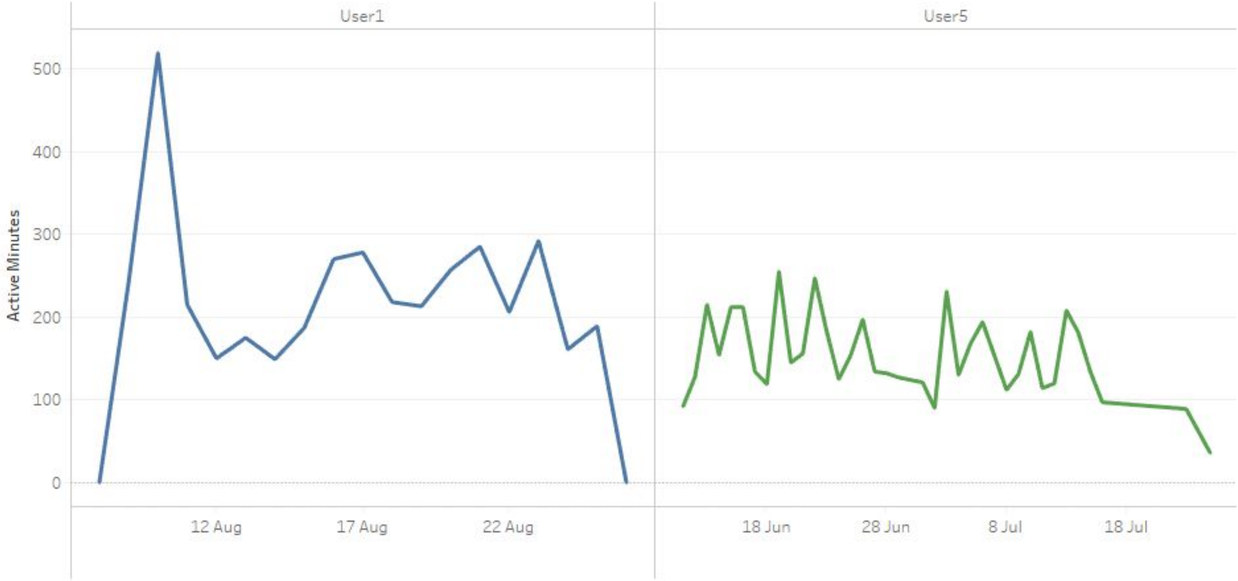

Source: Sample OnTrack usage data collected from healthy beta testers, June-August 2019

Reporting checklist for protocol of a clinical trial.

Based on the SPIRIT guidelines.

Instructions to authors

Complete this checklist by entering the page numbers from your manuscript where readers will find each of the items listed below.

Your article may not currently address all the items on the checklist. Please modify your text to include the missing information. If you are certain that an item does not apply, please write "n/a" and provide a short explanation.

Upload your completed checklist as an extra file when you submit to a journal.

In your methods section, say that you used the SPIRITreporting guidelines, and cite them as:

Chan A-W, Tetzlaff JM, Altman DG, Laupacis A, Gøtzsche PC, Krleža-Jerić K, Hróbjartsson A, Mann H, Dickersin K, Berlin J, Doré C, Parulekar W, Summerskill W, Groves T, Schulz K, Sox H, Rockhold FW, Rennie D, Moher D. SPIRIT 2013 Statement: Defining standard protocol items for clinical trials. Ann Intern Med. 2013;158(3):200-207

		Reporting Item	Page Number
**Administrative information**			
Title	#1	Descriptive title identifying the study design, population, interventions, and, if applicable, trial acronym	1
Trial registration	#2a	Trial identifier and registry name. If not yet registered, name of intended registry	2
Trial registration: data set	#2b	All items from the World Health Organization Trial Registration Data Set	n/a
Protocol version	#3	Date and version identifier	11
Funding	#4	Sources and types of financial, material, and other support	12
Roles and responsibilities: contributorship	#5a	Names, affiliations, and roles of protocol contributors	11

For peer review only

Roles and responsibilities: sponsor contact information	#5b	Name and contact information for the trial sponsor	n/a
Roles and responsibilities: sponsor and funder	#5c	Role of study sponsor and funders, if any, in study design; collection, management, analysis, and interpretation of data; writing of the report; and the decision to submit the report for publication, including whether they will have ultimate authority over any of these activities	11
Roles and responsibilities: committees	#5d	Composition, roles, and responsibilities of the coordinating centre, steering committee, endpoint adjudication committee, data management team, and other individuals or groups overseeing the trial, if applicable (see Item 21a for data monitoring committee)	n/a
**Introduction**			
Background and rationale	#6a	Description of research question and justification for undertaking the trial, including summary of relevant studies (published and unpublished) examining benefits and harms for each intervention	3
Background and rationale: choice of comparators	#6b	Explanation for choice of comparators	n/a
Objectives	#7	Specific objectives or hypotheses	4
Trial design	#8	Description of trial design including type of trial (eg, parallel group, crossover, factorial, single group), allocation ratio, and framework (eg, superiority, equivalence, non-inferiority, exploratory)	5
**Methods: Participants, interventions, and outcomes**			
Study setting	#9	Description of study settings (eg, community clinic, academic hospital) and list of countries where data will be collected. Reference to where list of study sites can be obtained	5
Eligibility criteria	#10	Inclusion and exclusion criteria for participants. If applicable, eligibility criteria for study centres and individuals who will	5

For peer review only

		perform the interventions (eg, surgeons, psychotherapists)	
Interventions: description	#11a	Interventions for each group with sufficient detail to allow replication, including how and when they will be administered	6
Interventions: modifications	#11b	Criteria for discontinuing or modifying allocated interventions for a given trial participant (eg, drug dose change in response to harms, participant request, or improving / worsening disease)	n/a
Interventions: adherance	#11c	Strategies to improve adherence to intervention protocols, and any procedures for monitoring adherence (eg, drug tablet return; laboratory tests)	n/a
Interventions: concomitant care	#11d	Relevant concomitant care and interventions that are permitted or prohibited during the trial	n/a
Outcomes	#12	Primary, secondary, and other outcomes, including the specific measurement variable (eg, systolic blood pressure), analysis metric (eg, change from baseline, final value, time to event), method of aggregation (eg, median, proportion), and time point for each outcome. Explanation of the clinical relevance of chosen efficacy and harm outcomes is strongly recommended	7
Participant timeline	#13	Time schedule of enrolment, interventions (including any run-ins and washouts), assessments, and visits for participants. A schematic diagram is highly recommended (see Figure)	Table 2
Sample size	#14	Estimated number of participants needed to achieve study objectives and how it was determined, including clinical and statistical assumptions supporting any sample size calculations	6
Recruitment	#15	Strategies for achieving adequate participant enrolment to reach target sample size	6
**Methods: Assignment of interventions (for controlled trials)**			
Allocation: sequence generation	#16a	Method of generating the allocation sequence (eg, computer-generated random numbers), and list of any factors for stratification. To reduce predictability of a random sequence, details of any planned restriction (eg, blocking) should be provided in a separate document that is unavailable to those who enrol participants or assign interventions	n/a

For peer review only

Allocation concealment mechanism	#16b	Mechanism of implementing the allocation sequence (eg, central telephone; sequentially numbered, opaque, sealed envelopes), describing any steps to conceal the sequence until interventions are assigned	n/a
Allocation: implementation	#16c	Who will generate the allocation sequence, who will enrol participants, and who will assign participants to interventions	n/a
Blinding (masking)	#17a	Who will be blinded after assignment to interventions (eg, trial participants, care providers, outcome assessors, data analysts), and how	n/a
Blinding (masking): emergency unblinding	#17b	If blinded, circumstances under which unblinding is permissible, and procedure for revealing a participant's allocated intervention during the trial	n/a
**Methods: Data collection, management, and analysis**			
Data collection plan	#18a	Plans for assessment and collection of outcome, baseline, and other trial data, including any related processes to promote data quality (eg, duplicate measurements, training of assessors) and a description of study instruments (eg, questionnaires, laboratory tests) along with their reliability and validity, if known. Reference to where data collection forms can be found, if not in the protocol	9
Data collection plan: retention	#18b	Plans to promote participant retention and complete follow-up, including list of any outcome data to be collected for participants who discontinue or deviate from intervention protocols	n/a
Data management	#19	Plans for data entry, coding, security, and storage, including any related processes to promote data quality (eg, double data entry; range checks for data values). Reference to where details of data management procedures can be found, if not in the protocol	9
Statistics: outcomes	#20a	Statistical methods for analysing primary and secondary outcomes. Reference to where other details of the statistical analysis plan can be found, if not in the protocol	9
Statistics: additional analyses	#20b	Methods for any additional analyses (eg, subgroup and adjusted analyses)	9

For peer review only

Statistics: analysis population and missing data	#20c	Definition of analysis population relating to protocol non-adherence (eg, as randomised analysis), and any statistical methods to handle missing data (eg, multiple imputation)	n/a
**Methods: Monitoring**			
Data monitoring: formal committee	#21a	Composition of data monitoring committee (DMC); summary of its role and reporting structure; statement of whether it is independent from the sponsor and competing interests; and reference to where further details about its charter can be found, if not in the protocol. Alternatively, an explanation of why a DMC is not needed	n/a
Data monitoring: interim analysis	#21b	Description of any interim analyses and stopping guidelines, including who will have access to these interim results and make the final decision to terminate the trial	n/a
Harms	#22	Plans for collecting, assessing, reporting, and managing solicited and spontaneously reported adverse events and other unintended effects of trial interventions or trial conduct	n/a
Auditing	#23	Frequency and procedures for auditing trial conduct, if any, and whether the process will be independent from investigators and the sponsor	n/a
**Ethics and dissemination**			
Research ethics approval	#24	Plans for seeking research ethics committee / institutional review board (REC / IRB) approval	10
Protocol amendments	#25	Plans for communicating important protocol modifications (eg, changes to eligibility criteria, outcomes, analyses) to relevant parties (eg, investigators, REC / IRBs, trial participants, trial registries, journals, regulators)	10
Consent or assent	#26a	Who will obtain informed consent or assent from potential trial participants or authorised surrogates, and how (see Item 32)	6
Consent or assent: ancillary studies	#26b	Additional consent provisions for collection and use of participant data and biological specimens in ancillary studies, if applicable	n/a
Confidentiality	#27	How personal information about potential and enrolled participants will be collected, shared, and maintained in order to protect confidentiality before, during, and after the trial	10

For peer review only

Declaration of interests	#28	Financial and other competing interests for principal investigators for the overall trial and each study site	12
Data access	#29	Statement of who will have access to the final trial dataset, and disclosure of contractual agreements that limit such access for investigators	10
Ancillary and post trial care	#30	Provisions, if any, for ancillary and post-trial care, and for compensation to those who suffer harm from trial participation	n/a
Dissemination policy: trial results	#31a	Plans for investigators and sponsor to communicate trial results to participants, healthcare professionals, the public, and other relevant groups (eg, via publication, reporting in results databases, or other data sharing arrangements), including any publication restrictions	10
Dissemination policy: authorship	#31b	Authorship eligibility guidelines and any intended use of professional writers	n/a
Dissemination policy: reproducible research	#31c	Plans, if any, for granting public access to the full protocol, participant-level dataset, and statistical code	n/a
**Appendices**			
Informed consent materials	#32	Model consent form and other related documentation given to participants and authorised surrogates	n/a
Biological specimens	#33	Plans for collection, laboratory evaluation, and storage of biological specimens for genetic or molecular analysis in the current trial and for future use in ancillary studies, if applicable	n/a

The SPIRIT checklist is distributed under the terms of the Creative Commons Attribution License CC-BY-ND 3.0. This checklist was completed on 07. October 2019 using https://www.goodreports.org/, a tool made by the EQUATOR Network in collaboration with Penelope.ai

For peer review only

BMJ Open

Protocol for a feasibility study of OnTrack: a digital system for upper-limb rehabilitation after stroke.

Journal:	*BMJ Open*
Manuscript ID	bmjopen-2019-034936.R2
Article Type:	Protocol
Date Submitted by the Author:	25-Feb-2020
Complete List of Authors:	Fusari, Gianpaolo; Royal College of Art, Helix Centre; Imperial College London, Helix Centre<br>Gibbs, Ella; Imperial College London, Helix Centre<br>Hoskin, Lily; Imperial College London, Helix Centre<br>Dickens, Daniel; Imperial College London, Helix Centre<br>Leis, Melanie; Imperial College London, Big Data and Analytical Unit, Institute of Global Health Innovation<br>Taylor, Elizabeth; Kingston and St George's Faculty of Health Social Care and Education<br>Jones, Fiona; Kingston and St George's Faculty of Health Social Care and Education<br>Darzi, Ara; Imperial College London, Helix Centre
**Primary Subject Heading**:	Neurology
Secondary Subject Heading:	Rehabilitation medicine, Neurology, Public health
Keywords:	Stroke < NEUROLOGY, REHABILITATION MEDICINE, NEUROLOGY, PUBLIC HEALTH

SCHOLARONE™
Manuscripts

I, the Submitting Author has the right to grant and does grant on behalf of all authors of the Work (as defined in the below author licence), an exclusive licence and/or a non-exclusive licence for contributions from authors who are: i) UK Crown employees; ii) where BMJ has agreed a CC-BY licence shall apply, and/or iii) in accordance with the terms applicable for US Federal Government officers or employees acting as part of their official duties; on a worldwide, perpetual, irrevocable, royalty-free basis to BMJ Publishing Group Ltd ("BMJ") its licensees and where the relevant Journal is co-owned by BMJ to the co-owners of the Journal, to publish the Work in this journal and any other BMJ products and to exploit all rights, as set out in our licence.

The Submitting Author accepts and understands that any supply made under these terms is made by BMJ to the Submitting Author unless you are acting as an employee on behalf of your employer or a postgraduate student of an affiliated institution which is paying any applicable article publishing charge ("APC") for Open Access articles. Where the Submitting Author wishes to make the Work available on an Open Access basis (and intends to pay the relevant APC), the terms of reuse of such Open Access shall be governed by a Creative Commons licence – details of these licences and which Creative Commons licence will apply to this Work are set out in our licence referred to above.

Other than as permitted in any relevant BMJ Author's Self Archiving Policies, I confirm this Work has not been accepted for publication elsewhere, is not being considered for publication elsewhere and does not duplicate material already published. I confirm all authors consent to publication of this Work and authorise the granting of this licence.

For peer review only

Title

Protocol for a feasibility study of OnTrack: a digital system for upper-limb rehabilitation after stroke.

Authors and affiliations

Gianpaolo Fusari[1], Ella Gibbs[1], Lily Hoskin[1], Daniel Dickens[1], Melanie Leis[2], Elizabeth Taylor[3], Fiona Jones[3], Ara Darzi[1]

[1]Helix Centre, Imperial College London and the Royal College of Art, London, United Kingdom
[2]Big Data and Analytical Unit, Institute of Global Health Innovation, Imperial College London, London, United Kingdom
[3]Kingston University and St George's, University of London, London, United Kingdom

Correspondence to:
Gianpaolo Fusari
HELIX Centre, Institute of Global Health Innovation
Imperial College London, 3rd Floor, Paterson Building, St Mary's Hospital, 16 South Wharf Road, London W2 1PF, UK
gianpaolo@helixcentre.com
+44 (0)77 6779 2770

Key words

Stroke, rehabilitation medicine, neurology, public health, digital health

Word count

4198

For peer review only

Abstract

Introduction

Arm weakness is a common problem after stroke (affecting 450,000 people in the UK) leading to loss of independence. Repetitive activity is critical for recovery but research shows people struggle with knowing what or how much to do, and keeping track of progress. Working with more than 100 therapists (occupational therapists and physiotherapists) and patients with stroke, we co-developed the OnTrack intervention - consisting of software for smart-devices and coaching support - that has the potential to address this problem. This is a protocol to assess the feasibility of OnTrack for evaluation in a randomised control trial.

Methods and analysis

A mixed methods, single-arm study design will be used to evaluate the feasibility of OnTrack for hospital and community use. A minimum sample of 12 participants from a stroke unit will be involved in the study for 14 weeks. During week 1, 8 and 14 participants will complete assessments relating to their arm function, arm impairment, and activation. During weeks 2-13 participants will use OnTrack to track their arm movement in real time, receive motivational messages, and face-to-face sessions to address problems, gain feedback on activity, and receive self-management skills coaching. All equipment will be loaned to study participants. A parallel process evaluation will be conducted to assess the intervention's fidelity, dose and reach, using a mixed methods approach. A Public and Patient Involvement (PPI) group will oversee the study and help with interpretation and dissemination of qualitative and quantitative data findings.

Ethics and dissemination

Ethical approval granted by the NHS Health Research Authority, Health and Care Research Wales, and the London - Surrey Research Ethics Committee (ref.19/LO/0881). Trial results will be submitted for publication in peer review journals, presented at international conferences and disseminated amongst stroke communities. The results of this trial will inform development of a definitive trial.

Trial registration details

ClinicalTrials.gov (NCT03944486), pre-results.

For peer review only

Strengths and limitations

- This is a feasibility trial of a novel intervention which employs an integrated approach for tracking arm activity and coaching with the aim of increasing stroke survivors' confidence and ability to use their impaired arm in daily activities, increasing the opportunities for repetitive rehabilitation (repeating a movement or series of movements with a rehabilitative or functional aim).
- Patient and Public Involvement (PPI) with more than 100 stroke survivors, carers, and clinicians have contributed to our needs-finding phase, co-designed OnTrack and informed the feasibility study. A new PPI group will oversee the running of the study and help with interpretation of qualitative and quantitative data findings.
- An independent process evaluation will provide detailed information about implementation, context, and the mechanisms of impact of the intervention. Findings will help in the understanding of intervention fidelity and training needs required for a definitive trial.
- For pragmatic reasons the study uses a non-randomised design carried out at a single site- this will limit understanding about randomisation and recruitment
- Participants will not be followed-up after intervention period; however participant views will be sought regarding appropriate follow-up times in a subsequent definitive trial.

Introduction

Every year around the world over 15 million people experience a stroke, leaving 5 million people with a permanent disability.(1) Stroke is the leading cause of disability in the UK; half of the nearly 1.2 million stroke survivors who live in the country have some form of disability, significantly contributing to the loss of independence and feeling of isolation that they experience. (2),(3) Furthermore, stroke is estimated to cost UK society £26 billion every year, with the vast majority of these costs borne by the informal care sector.(2)

Upper-limb (arm) weakness is the main cause of physical impairment affecting 75% of disabled stroke survivors; this equates to around 450,000 people in the UK.(2) Dose-intensive repetitive rehabilitation is widely accepted as the 'gold-standard' for regaining ability after stroke, however, NHS resources are often limited and unable to provide this.(4) A recent Cochrane review of over 500 trials failed to yield high-quality practice recommendations for interventions for the upper-limb.(5) Arm recovery after stroke is a national research priority.(6) There is a correlation between physical activity after stroke and the ability to perform activities of daily living (most of which involve the use of the arm).(7) Despite this evidence, studies suggest that the actual time patients are active is minimal.(8) Many current approaches to increasing repetitive rehabilitation focus on improving the prescribed rehabilitation sessions (typically lasting 45-60 minutes), often by employing gamification techniques.(9,10)

For peer review only

Whilst this is important, there is untapped potential to increase repetitive rehabilitation by targeting the large proportion of the day where patients are going about their daily activities and can use their arm movement (however small) to a greater extent. Capacity for activity could be increased further by using self-management methods as demonstrated by several different programmes in stroke and other long-term conditions.(11-14) This has informed the development of OnTrack which aims to increase opportunities for activity by improving individuals' self-management skills through tailored support and real-time activity feedback on their arm movement.

An unpublished ethnographic study conducted by the Helix Centre (funded by Innovate UK) confirmed what other studies have shown (7,8,15) that patients struggle to see and keep track of improvements, this impacts their motivation and leaves them dependent on therapists for feedback. Stroke survivors often report feeling unsupported after leaving hospital and not knowing how to best help themselves improve their arm function.(16-18) Feedback gathered from over 100 stroke survivors and clinicians was the basis for developing the OnTrack intervention.

A proof-of-concept test of OnTrack gathered data from a small group of patients (n=7) and confirmed that the intervention was safe and generally users could understand how and when to use it. Participants reported they were more aware of their impaired arm and had increased confidence in using it for new tasks. A 20% mean increase in minutes of activity on the impaired arm was observed. The work conducted to date is unpublished and has some limitations however it has shaped the intervention and suggests that OnTrack has the potential to be a scalable solution that requires minimal training and could be used in conjunction with NHS services to help increase the overall amount of activity performed with the impaired arm. This study will assess the feasibility of the OnTrack intervention and inform the design of a definitive randomised controlled trial (RCT) to evaluate its clinical effectiveness, and follows the Standard Protocol Items: Recommendations for Interventional Trials (SPIRIT) guidelines.(19)

Methods and analysis

Aims and objectives

The primary aim is to evaluate the feasibility of an RCT to test the effectiveness of the OnTrack intervention for upper limb rehabilitation after stroke.

The objectives are to:

- Assess the feasibility of recruitment from hyper-acute and acute stroke units, and rehabilitation wards to ascertain strategy and recruitment rates.
- Assess dropout rates by observing adherence and compliance with the intervention.
- Understand the acceptability and usability of the intervention by stroke survivors.
- Understand the acceptability of study procedures by healthcare professionals.
- Explore implementation fidelity, dose and reach of the OnTrack intervention.

For peer review only

The study will also collect clinical outcomes regarding arm function, impairment and activation to identify an appropriate primary outcome, and to estimate parameters for a sample size calculation for an RCT (Table 1).

Study design

A feasibility study with a nested process evaluation (Figure 1). The study is a single-site, non-randomised intervention trial. The design of the study was developed through a collaborative approach between the study researchers, a PPI steering group, front-line therapists, and the Research Design Service at the National Institute for Health Research.

An independent process evaluation will be conducted in parallel to learn about usage and engagement mechanisms of participants, therapists and other frontline staff, providing critical information for implementation fidelity and impact mechanisms necessary for scale-up.

Study setting

The study will be conducted at an inner city NHS hospital Trust in London. Recruited participants will be able to continue to receive the intervention at home if discharged from hospital prior to ending the intervention period (14 weeks).

Participants

The inclusion criteria encompasses:

- Adults (aged 18 or over).
- Stroke diagnosis less than 6 months previously (first or recurrent). Some participants will be recruited from an in-patient rehabilitation ward, hence the 6 month post-stroke limit.
- Arm impairment of any type or level (including weakness - including dense hemiplegia, neglect, and sensory deficits). This to enable better understanding of which impairment level groups could benefit *or not* from using the intervention, especially considering the impact it may have on people's motivation regardless of their level of impairment.
- Ability to provide informed consent.
- Reliability to communicate (verbally or nonverbally) and understand English.
- Ability to read a predefined short message.

Potential participants who at the time of recruitment (or during participation) present with any of the following will be excluded:

- Unstable medical condition.
- Self reported "severe" pain in the arm affected either at rest or during movement.
- Severe oedema in the arm affected by their stroke, judged by the consenting therapist.
- Known discharge plans to a hospital other than the site Trust or residential care in less than 7 weeks (a small proportion of patients staying at CNRU may be in hospital for up to 12 weeks).

For peer review only - http://bmjopen.bmj.com/site/about/guidelines.xhtml

- Participants who are unable to engage with the intervention for a period of more than 7 consecutive days will be reviewed in a case-by-case basis by the members of the team responsible for delivering the intervention to determine if study continuation is appropriate

Recruitment

Participants will be recruited from the Hyperacute Stroke Unit (HASU), Acute Stroke Unit (ASU), and Clinical Neurorehabilitation Unit (CNRU) at an inner city NHS hospital Trust in London.

Stroke therapists (occupational therapists, physiotherapists) will be responsible for screening and identifying suitable patients. They will introduce the study to potential participants and provide information documents. Potential participants will be given a minimum of 24 hours to consider the advantages and disadvantages of participating in the study and to formulate questions. Therapists will be able to answer questions or will liaise with the research team to provide an answer. Once all questions are answered and a potential participant is willing to participate, consent will be taken by the therapist. Only at this stage will patient information be shared with the research team. There may be situations where a therapist is only able to take verbal consent from a participant due to time or material constraints, in such cases the researchers will be able to take written consent from the participant upon first meeting them.

Sample size calculation

Guidelines advocate a sample size of 12-30 participants for feasibility studies.(20) Experienced clinical academics and clinicians at the trial site have advised to expect about 50% of eligible patients to agree to participation and a 50% completion rate. This has informed a recruitment plan to identify at least 60 potential participants in a period of 30 weeks to reach the minimum sample size.

Intervention

The intervention is the OnTrack system as a whole. The system consists of smart-devices (smartphone and smartwatch), software (OnTrack app), and coaching support. Smart-devices are used to track arm movement. Motivational messages and a real-time display of completed arm activity (in minutes) are presented to the user via the OnTrack app. Coaching support is provided through fortnightly consultations by the researchers. During consultations, participants will receive self-management training informed by the Bridges Self-Management (21) and TaCAS (22) self-management programs. Coaching sessions are themed around principles of self-management (see Table 2, OnTrack Consultation column).

Data gathered by the OnTrack system can be accessed by the researchers via a digital dashboard to inform consultations.

For peer review only - http://bmjopen.bmj.com/site/about/guidelines.xhtml

Participants will be loaned all equipment necessary for the trial and no previous experience with using smart-devices is required to participate. Technical support will be provided only in cases where the hardware and/or software fail to perform the required functions to deliver the intervention.

Table 2 provides a participation schedule and a summary of the intervention procedures.

Outcomes

Feasibility of trial design and procedures

- Recruitment strategy and rates (feasibility of recruitment from HASU, ASU, CNRU wards) - percentage of patients: screened; eligible; approached; consented; excluded after screening. Participants consented and recruited will be logged by the research team in DOCUMAS(23)
- Compliance and adherence to intervention - measure of minutes of activity per participant as recorded by the OnTrack app, engagement with OnTrack app as measured by system analytics (for example: compliance with starting tracking arm activity daily, number of times and times of the day a particular screen is visited, the number of messages read and replied to, etc.)
- Completion rates - percentage of participants who complete the 14-week intervention period (not dropping out or being withdrawn from the study)
- Acceptability and reasons for decline/withdrawal - number of participants who withdraw or decline the intervention and reasons why. A record of reasons for withdrawal and declining will be kept by the researchers. Reasons will be categorised in order of most common; this information will help the research team understand the reasons why someone might drop out or decline to participate in the study.

Clinical assessments

As a secondary objective, clinical outcomes will be collected at different time points by a qualified member of the research team to identify an appropriate primary outcome, and to estimate parameters for a sample size calculation for an RCT (Table 1). The outcome measures and assessments are listed below.

Patient activation

Patient activation is a concept recognised by the NHS that describes the knowledge, skills and confidence a person has in managing their own health and health care.(24) This will be measured using the Patient Activation Measure (PAM)(25) which has been validated in stroke populations in the UK.(26) The PAM survey measures patients on a 0–100 scale and can categorise patients into one of four activation levels along an empirically derived continuum.(25) Activation levels will be used to allocate participants one of three different OnTrack coaching tiers. The tiers aim to make the different aspects of the coaching more relevant and meaningful for the individual participant and their stage of recovery and self-management.

For peer review only

Arm impairment

Arm impairment will be measured objectively using the Fugl-Meyer Assessment for upper extremity (FMA-UE).(27) The FMA-UE has been tested extensively, and is found to have excellent psychometric properties and is recommended as core measures to be used in every stroke recovery and rehabilitation trial.(28)

Arm function

Arm function will be assessed using the Upper-Extremity Motor Activity Log-14 (MAL).(29) The MAL is a scripted, structured interview developed to self-report the amount and quality of use of the impaired arm in individuals with stroke in 14 different activities of daily living.

Gross level of disability

The modified Rankin Scale (mRS)(30) is the most prevalent functional outcome measure in contemporary stroke trials. The mRS quantifies disability using an ordinal hierarchical grading from zero (no symptoms) to 5 (severe disability).

Arm pain

Pain will be assessed using a visual analogue scale (VAS) from 0 (no pain) to 10 (excruciating pain) over the last 24 hours. VAS is a valid measure of pain intensity and is responsive to change.(31) Individuals scoring 3/10 or more in the affected arm will be withdrawn from the study unless their pain is only on movements that are not part of their usual everyday activities (e.g. arm pain when doing overhead reaching).

Cognitive impairment

Cognitive impairment will be assessed using the Montreal Cognitive Assessment (MoCA). The MoCA is a brief cognitive screening tool with high sensitivity and specificity for detecting mild cognitive impairment.(32) The MoCA defines impairment as follows: score of 18-25 = mild, 10-17 = moderate, <10 = severe.(32) Participants' scores will be used to look for associations between the use of OnTrack and any cognitive impairment.

Perceptual neglect

Albert's Test (AT) is being used to assess for unilateral spatial neglect (USN). This a simple test where participants are asked to cross out lines ruled in a standard fashion on a sheet of paper. If any lines are left uncrossed, and more than 70% of uncrossed lines are on the same side as motor deficit, USN is indicated. This may be quantified in terms of the percentage of lines left uncrossed. The test is very easy to administer and is a good predictor of functional activity six months after stroke onset.(33)

For peer review only

Quality of life

The EQ-5D-5L is a widely used standardised preference based measure of health status developed by the EuroQol Group in order to provide a simple, generic measure of health for clinical and economic appraisal.(34)

Additional assessments

A Lap-to-Table (LTT) timed test will be performed where the researchers measure the time it takes a participant to move their hand three times from resting on their lap to a table positioned in front of them. This test is performed to assess its potential to use as part of the inclusion criteria for an RCT.

The NHS Friends and Family Test (FFT)(35) will be used to obtain feedback on the overall experience of using OnTrack and participating in the trial. Participants will be asked: “How likely are you to recommend OnTrack to friends and family if they needed similar care or treatment?” with answers provided in a Likert 5-point scale ranging from “extremely likely” to “extremely unlikely” and an “I don’t know” option.

The System Usability Scale (SUS) will be used to subjectively assess the usability of the OnTrack intervention. The test is a simple, ten-item scale covering a variety of aspects of system usability, such as the need for support, training, and complexity, and thus have a high level of face validity for measuring the usability of a system.(36)

Process evaluation

A process evaluation will be carried out by researchers working independently to the intervention team and in parallel to the trial to determine whether the OnTrack intervention was delivered as intended and to understand the mechanisms of impact. The aim of the process evaluation at the feasibility stage is mainly to understand how the trial design and intervention could be optimised ahead of an RCT.(37) A logic model(38,39) that defines the intervention in terms of inputs, outputs, causal assumptions and expected outcomes has been developed to help identify core questions for the evaluation team to explore (Figure 2). The evaluation team will observe 10% of all intervention sessions with the objective of documenting fidelity, dose and reach of the intervention.

Critical reflection and the process evaluation will help refine the intervention, as shown by mid-range theories (i.e. theories that help understand implementation).(40) Interim results will be shared with the intervention team at the half-way point with the objective to review some of the procedures and make minor adjustments as necessary.

In-depth semi-structured interviews will be conducted with patients at the end of their participation, a minimum sample of 12 is anticipated. A topic guide with themes drawing from the logic model will be used. Interviews will focus on participants’ experiences using OnTrack, their perceptions of arm tracking, motivational messaging and the researcher consultations. Additionally, the interviews will explore participants’ perceptions of the impact OnTrack had on them in terms of progress, awareness, participation, and confidence in self-

For peer review only

management. Participants' responses will be compared against activity data collected from the OnTrack app.

NHS therapists caring for participants taking part will be consented and invited to complete a short online survey to gather their feedback regarding acceptability of study procedures, they have the option to respond anonymously. The total number of therapists involved is difficult to predict as there may be team changes and staff movement during the course of the study. The survey will ask questions around three themes: 1) participation, relevance, quality and time spent in study procedures; 2) opinions on the benefit/detriment OnTrack may have for patients; 3) opinions on how the intervention may or may not fit with service provision and their workflow.

Data analysis

Analysis will be completed on the parameters and implementation of the study in addition to the usability of OnTrack.

Data collected for the process evaluation will be a combination of qualitative data from interviews with stroke participants and therapists to explore their experiences of using OnTrack, as well as quantitative data on usage of OnTrack and the self-reported SUS. OnTrack therapy support sessions will be monitored through a fidelity checklist and observations (10 live sessions will be observed in total. In addition, the evaluation team will have access to recorded sessions that can be observed at their discretion). Interview data will undergo thematic analysis by the evaluation team. Data will be entered into NVIVO,(41) line by line coding and analysis will be informed by Braun and Clark's approach to thematic analysis.(42)

Changes over time will be evaluated in both OnTrack usage and outcome measures.

For OnTrack usage, the team will analyse users' activity patterns by day and hour of day. Figure 3 illustrates examples of visualisations created using aggregated data captured by OnTrack from healthy beta testers between June-August 2019. It compares users on active minutes per hour of day (aggregated over time) and active minutes per day.

OnTrack also captures specific usage metrics, including:

- Number of times OnTrack messages were opened
- Number of times daily and weekly activity were viewed on the phone
- Number of swipes on watch to reveal activity graph

For each patient, we will plot the values above against their minutes of activity to better understand the potential impact of the app on activity over time.

The self-reported PAM will be captured at weeks 1, 8 and 14 for each user. It will be analysed in relation to the minutes of activity of each user over time to better understand the potential impact of the app on their levels of activation. SUS will be captured at weeks 8 and 14 and will be compared against actual usage metrics (described above) to assess usability.

For peer review only

Whilst conducting meaningful significant subgroup analyses would be difficult given the relatively small sample size, we believe that outputs from this study could potentially inform the subgroups that might be considered for inclusion in a larger trial.

All data will be stored and accessed in accordance with GDPR guidance.

Clinical trial support will be provided by the Big Data and Analytical Unit (BDAU) at Imperial College London's Institute of Global Health Innovation (IGHI).

Patient and public involvement (PPI)

To date, over 100 stroke survivors, carers and therapists have been involved in the design of OnTrack. Participants and have been instrumental in highlighting areas for improvement in upper-limb stroke rehabilitation. They have contributed to a co-design process (including workshops, interviews, observations and surveys) resulting in the design, development and initial testing of OnTrack.

A steering group comprising of four stroke survivors was formed for the purpose of this feasibility study. Diversity within the group - both in terms of demographics and stroke severity - was considered. The group has supervised the development of all patient-facing material ensuring its clarity. They will also participate in data analysis by helping to refine themes and key messages arising from qualitative interviews. Participants will be trained by experienced researchers for this purpose.

The steering group will meet five times over the duration of the study, including an initial briefing session at the start to outline their involvement. Steering group members will be key members of the research team and their time and travel will be reimbursed according to INVOLVE(43) guidelines.

The PPI involvement plan was shared with Imperial College London's PPI 'Research Partners Group' on 21.02.19 who felt that the needs of the steering group have been accounted for.

Ethics and dissemination

The OnTrack study will be conducted in accordance with the recommendations for physicians involved in research on human subjects adopted by the 18th World Medical Assembly, Helsinki 1964 and later revisions; and in compliance with the relevant UK and European legislation including the NHS Health Research Authority (HRA) policy frameworks and the General Data Protection Regulation 2018 (GDPR).

The study was granted ethical approval by the HRA, Health and Care Research Wales, and the London - Surrey Research Ethics Committee (ref. 19/LO/0881). Local site capacity and capability approval has been granted by the hospital Trust.

For peer review only

The current approved protocol version is V1.3 dated 19.06.2019. Protocol amendments will be submitted for approval to the NHS HRA in the first instance and to the local site thereafter ahead of implementation.

The Chief Investigator is responsible for preserving the confidentiality of participants taking part in the study. Researchers will have patients' names, contact numbers, emails and home addresses for the purposes of arranging visits. This information will be stored in accordance with GDPR legislation. Participants are free to withdraw from the study at any time. However, anonymised activity data collected may still be used for data analysis as this is unlinked of any patient identifiable information.

The day-to-day management of the study will be coordinated by the Helix Centre. A study steering committee formed by the intervention team, evaluation team, PPI group, and representatives from the local site will meet at regular intervals throughout the study.

Regular updates about the trial will be made available through social media, blog posts, newsletters and the Helix Centre website (www.helixcentre.com). Trial results will be submitted for publication in journals, presented at national and international stroke meetings and conferences and disseminated amongst stroke communities.

Trial status

The first participant was enrolled on 09.09.2019 and recruitment is expected to complete by the end of March 2020. Enrolment and data collection was continuing as planned at the time of submission of this protocol.

Author statement

AD is grant holder and has project oversight along with DD. GF, EG and LH developed the intervention and conceived of the study. GF, EG, and FJ initiated the study design and ET and ML helped with further refinement. EG and GF are responsible for delivering the intervention and data collection. FJ and ET are responsible for the process evaluation. ML provides statistical expertise in trial design and is conducting the primary statistical analysis. All authors contributed to the refinement of the study protocol and approved the final manuscript.

Funding

This study is funded by the NIHR Imperial Biomedical Research Centre (BRC), grant 1215-20013. The views expressed are those of the author(s) and not necessarily those of the NIHR or the Department of Health and Social Care.

All intellectual property associated with the OnTrack system is owned by the Helix Centre which is a collaboration between Imperial College London and the Royal College of Art.

For peer review only

Data statement

Anonymised data will be made available in a public repository once the data have obtained validation through publication.

Conflict of interests

FJ is the founder of the social enterprise Bridges Self-Management. She has not received any financial support for this work that could have influenced the design.

Acknowledgments

The authors would like to acknowledge the contributions of the members of the PPI group; Jennifer Crow and Sarah Daniels for their input on recruitment and sample size calculations; and Dr Gaby Judah for her help in defining some aspects of the intervention.

Figure legends

Figure 1

Trial diagram

Figure 2

Logic model

Figure 3

Examples of visualisations created using aggregated data captured by OnTrack from healthy beta testers. Data for a minimum of 5 and a maximum of 18 days were aggregated for the period between June and August 2019.

For peer review only

Table 1

Outcome measures

Concept	Assessment	Week of administration
Patient Activation / Engagement	Patient Activation Measure (PAM)	1, 8, 14
Arm impairment	Fugl-Meyer Assessment for upper extremity (FMA-UE)	1, 8, 14
Arm function	Upper-Extremity Motor Activity Log-14 (MAL)	1, 8, 14
Gross level of disability	modified Rankin Scale (mRS)	1, 8, 14
Arm pain	Visual Analogue Scale (VAS)	1, 8, 14
Cognitive impairment	Montreal Cognitive Assessment (MoCA)	1, 8, 14
Arm neglect	Albert's Test (AT)	1, 8, 14
Quality of life	EQ-5D-5L	1, 8, 14
Arm function	Lap-to-Table (LTT)	1, 8, 14
Service experience	Friends and Family Test (FTT)	8, 14
System usability	System Usability Scale (SUS)	14

For peer review only

Table 2

Intervention and Participation schedule

Week	Phase	Description	OnTrack consultation	Assessments
0	Information and consent	NHS therapists screen for eligible patients, provide information and consent participants		Screening, information, and consent
1	Baseline assessment (initial)	Participants complete outcome measures and wear activity trackers (Axivity AX3) on both arms during waking hours (typically 12 hours per day) for one week to gather accelerometer data which is translated into minutes of activity. This data creates a baseline of activity allowing left-to-right arm usage comparison		PAM, FMA-UE, MAL, mRS, VAS, MoCA, AT, EQ-5D-5L, LTT
2	OnTrack intervention	Participants wear a smartwatch (Apple Watch Series 3 or 4) on their impaired arm only during waking hours (typically 12 hours per day). They will receive real-time feedback on the amount of movement completed (measured in minutes) and daily motivational messages. Participants will receive fortnightly consultations with a researcher to troubleshoot and receive self-management skills training<br><br>Baseline assessments are repeated during week 8 (halfway)	Onboarding	
3			Check-in & self-management skills training (Problem Solving)	
4				
5			Check-in & self-management skills training (Self-Discovery)	
6				
7			Check-in & self-management skills training (Goal Setting)	
8			Halfway assessment<br><br>Check-in & self-management skills training (Goal Setting	PAM, FMA-UE, MAL, mRS, VAS, MoCA, AT, EQ-5D-5L, LTT, FFT

For peer review only

			cont.)	
9				
10			Check-in & self-management skills training (Reflection)	
11				
12			Check-in & self-management skills training (Sign-posting)	
13				
14	Baseline assessment (exit)	Participants complete outcome measures and wear activity trackers (Axivity AX3) on both arms during waking hours (typically 12 hours per day) for one week to gather accelerometer data which is translated into minutes of activity. This data creates a baseline of activity allowing left-to-right arm usage comparison		PAM, FMA-UE, MAL, mRS, VAS, MoCA, AT, EQ-5D-5L, LTT, FFT, SUS
15	Feedback	Independent evaluator leads feedback sessions with participants who have completed the intervention<br><br>End of participation		Semi-structured interview, online survey (therapists)

For peer review only

References

References
(1) WHO. *The Atlas of Heart Disease and Stroke.* Available from: https://www.who.int/cardiovascular_diseases/resources/atlas/en/ [Accessed 04 Oct 2019].
(2) Stroke Association. *State of the Nation: stroke statistics.* Stroke Association. 2018.
(3) Salter K, Hellings C, Foley N, Teasell R. The Experience of Living with Stroke: A Qualitative Meta-Synthesis. *Journal of Rehabilitation Medicine.* 2008; 40 (8): 595-602. Available from: doi: 10.2340/16501977-0238 Available from: http://www.ingentaconnect.com/content/mjl/sreh/2008/00000040/00000008/art00003 .
(4) Care Quality Commission. *Supporting life after stroke: a review of services for people who have had a stroke and their carers.* London: Care Quality Commission. 2011.
(5) Pollock A, Farmer SE, Brady MC, Langhorne P, Mead GE, Mehrholz J, et al. Interventions for improving upper limb function after stroke. *The Cochrane database of systematic reviews.* 2014; 2014 (11): CD010820. Available from: doi: 10.1002/14651858.CD010820.pub2 Available from: https://www.ncbi.nlm.nih.gov/pubmed/25387001 .
(6) Pollock A, St George B, Fenton M, Firkins L. Top 10 Research Priorities Relating to Life after Stroke – Consensus from Stroke Survivors, Caregivers, and Health Professionals. *International Journal of Stroke.* 2014; 9 (3): 313-320. Available from: doi: 10.1111/j.1747-4949.2012.00942.x Available from: https://journals.sagepub.com/doi/full/10.1111/j.1747-4949.2012.00942.x .
(7) Kunkel D, Fitton C, Burnett M, Ashburn A. Physical inactivity post-stroke: a 3-year longitudinal study. *Disability & Rehabilitation.* 2015; 37 (4): 304-310. Available from: doi: 10.3109/09638288.2014.918190 Available from: http://www.tandfonline.com/doi/abs/10.3109/09638288.2014.918190 .
(8) Bernhardt J, Dewey H, Thrift A, Donnan G. Inactive and Alone: Physical Activity Within the First 14 Days of Acute Stroke Unit Care. *Stroke.* 2004; 35 (4): 1005-1009. Available from: doi: 10.1161/01.STR.0000120727.40792.40 Available from: http://stroke.ahajournals.org/cgi/content/abstract/35/4/1005 .
(9) Laver KE, Lange B, George S, Deutsch JE, Saposnik G, Crotty M. Virtual reality for stroke rehabilitation. *The Cochrane database of systematic reviews.* 2017; 11 (11): CD008349. Available from: doi: 10.1002/14651858.CD008349.pub4 Available from: https://www.ncbi.nlm.nih.gov/pubmed/29156493 .
(10) Loureiro R, Harwin W, Nagai K, Johnson M. Advances in upper limb stroke rehabilitation: a technology push. *Medical & Biological Engineering & Computing.* 2011; 49 (10): 1103-1118. Available from: doi: 10.1007/s11517-011-0797-0 Available from: https://www.ncbi.nlm.nih.gov/pubmed/21773806 .
(11) Jones F, Gage H, Drummond A, Bhalla A, Grant R, Lennon S, et al. Feasibility study of an integrated stroke self-management programme: a cluster-randomised controlled trial. *BMJ Open.* 2016; 6 (1): e008900. Available from: doi: 10.1136/bmjopen-2015-008900 Available from: http://dx.doi.org/10.1136/bmjopen-2015-008900 .
(12) Harwood M, Weatherall M, Talemaitoga A, Barber PA, Gommans J, Taylor W, et al. Taking charge after stroke: promoting self-directed rehabilitation to improve quality of life – a randomized controlled trial. *Clinical Rehabilitation.* 2012; 26 (6): 493-501. Available from: doi: 10.1177/0269215511426017 Available from: https://journals.sagepub.com/doi/full/10.1177/0269215511426017 .
(13) Kendall E, Catalano T, Kuipers P, Posner N, Buys N, Charker J. Recovery following stroke: The role of self-management education. *Social Science & Medicine.* 2007; 64 (3): 735-746. Available from: doi: 10.1016/j.socscimed.2006.09.012 Available from: https://www.sciencedirect.com/science/article/pii/S0277953606004801 .
(14) Grady PA, Gough LL. Self-Management: A Comprehensive Approach to Management of Chronic Conditions. *American journal of public health.* 2014; 104 (8): e25-e31. Available from:

doi: 10.2105/AJPH.2014.302041 Available from: https://www.ncbi.nlm.nih.gov/pubmed/24922170 .
(15) Esmonde T, McGinley J, Wittwer J, Goldie P, Martin C. Stroke rehabilitation: patient activity during non-therapy time. *Australian Journal of Physiotherapy.* 1997; 43 (1): 43-51. Available from: doi: 10.1016/S0004-9514(14)60401-3 Available from: https://www.sciencedirect.com/science/article/pii/S0004951414604013 .
(16) Buckland D. Leaving hospital is 'like falling off a cliff' for stroke survivors. *Raconteur.* APRIL 27, 2017 . Available from: https://www.raconteur.net/healthcare/leaving-hospital-is-like-falling-off-a-cliff-for-stroke-survivors [Accessed Oct 04 2019].
(17) McKevitt C, Fudge N, Redfern J, Sheldenkar A, Crichton S, Rudd AR, et al. Self-Reported Long-Term Needs After Stroke. *Stroke.* 2011; 42 (5): 1398-1403. Available from: doi: 10.1161/STROKEAHA.110.598839 Available from: https://www.ncbi.nlm.nih.gov/pubmed/21441153 .
(18) Woodman P, Riazi A, Pereira C, Jones F. Social participation post stroke: a meta-ethnographic review of the experiences and views of community-dwelling stroke survivors. *Disability & Rehabilitation.* 2014; 36 (24): 2031-2043. Available from: doi: 10.3109/09638288.2014.887796 Available from: http://www.tandfonline.com/doi/abs/10.3109/09638288.2014.887796 .
(19) Chan A, Tetzlaff JM, Altman DG, Laupacis A, Gøtzsche PC, Krleža-Jerić K, et al. SPIRIT 2013 Statement: Defining Standard Protocol Items for Clinical Trials. *Annals of internal medicine.* 2013; 158 (3): 200-207. Available from: doi: 10.7326/0003-4819-158-3-201302050-00583 Available from: https://www.ncbi.nlm.nih.gov/pubmed/23295957 .
(20) Billingham SAM, Whitehead AL, Julious SA. An audit of sample sizes for pilot and feasibility trials being undertaken in the United Kingdom registered in the United Kingdom Clinical Research Network database. *BMC medical research methodology.* 2013; 13 (1): 104. Available from: doi: 10.1186/1471-2288-13-104 Available from: https://www.ncbi.nlm.nih.gov/pubmed/23961782 .
(21) Bridges Self-Management Limited. *Bridges Self-Management.* Available from: http://www.bridgesselfmanagement.org.uk/ [Accessed 07 Oct 2019].
(22) Medical Research Institute of New Zealand. *Taking Charge After Stroke (TaCAS).* Available from: https://mrinz.ac.nz/programmes/stroke/ [Accessed 07 oct 2019].
(23) DOCUMAS. *DOCUMAS: efficient health research management.* Available from: https://www.documas.eu/ [Accessed 04 Oct 2019].
(24) Hibbard J, Gilburt H. *Supporting people to manage their health An introduction to patient activation.* London: The Kings Fund. 2014.
(25) Hibbard JH, Stockard J, Mahoney ER, Tusler M. Development of the Patient Activation Measure (PAM): Conceptualizing and Measuring Activation in Patients and Consumers. *Health Services Research.* 2004; 39 (4p1): 1005-1026. Available from: doi: 10.1111/j.1475-6773.2004.00269.x Available from: https://onlinelibrary.wiley.com/doi/abs/10.1111/j.1475-6773.2004.00269.x .
(26) Roberts NJ, Kidd L, Dougall N, Patel IS, McNarry S, Nixon C. Measuring patient activation: The utility of the Patient Activation Measure within a UK context—Results from four exemplar studies and potential future applications. *Patient Education and Counseling.* 2016; 99 (10): 1739-1746. Available from: doi: 10.1016/j.pec.2016.05.006 Available from: https://www.clinicalkey.es/playcontent/1-s2.0-S0738399116301938 .
(27) Sahlgrenska Academy Institute of Neuroscience and Physiology, University of Gothenburg. *Fugl-Meyer.* Available from: https://neurophys.gu.se/english/departments/clinical_neuroscience_and_rehabilitation/rehabilitation-medicine/fugl-meyer [Accessed 04 Oct 2019].
(28) Pamela W Duncan, Martha Propst, Steven G Nelson. Reliability of the Fugl-Meyer Assessment of Sensorimotor Recovery Following Cerebrovascular Accident. *Physical Therapy.*

For peer review only

1983; 63 (10): 1606-1610. Available from: doi: 10.1093/ptj/63.10.1606 Available from: http://ptjournal.apta.org/content/63/10/1606.abstract .
(29) Uswatte G, Taub E, Morris D, Vignolo M, McCulloch K. Reliability and Validity of the Upper-Extremity Motor Activity Log-14 for Measuring Real-World Arm Use. *Stroke.* 2005; 36 (11): 2493-2496. Available from: doi: 10.1161/01.STR.0000185928.90848.2e Available from: http://stroke.ahajournals.org/cgi/content/abstract/36/11/2493 .
(30) Quinn TJ, Dawson J, Walters MR, Lees KR. Exploring the Reliability of the Modified Rankin Scale. *Stroke.* 2009; 40 (3): 762-766. Available from: doi: 10.1161/STROKEAHA.108.522516 Available from: http://stroke.ahajournals.org/cgi/content/abstract/40/3/762 .
(31) Jensen MP, Karoly P, Braver S. The measurement of clinical pain intensity: a comparison of six methods. *Pain.* 1986; 27 (1): 117-126. Available from: doi: 10.1016/0304-3959(86)90228-9 Available from: https://www.sciencedirect.com/science/article/pii/0304395986902289 .
(32) Nasreddine ZS, Phillips NA, Bédirian V, Charbonneau S, Whitehead V, Collin I, et al. The Montreal Cognitive Assessment, MoCA: A Brief Screening Tool For Mild Cognitive Impairment. *Journal of the American Geriatrics Society.* 2005; 53 (4): 695-699. Available from: doi: 10.1111/j.1532-5415.2005.53221.x Available from: https://onlinelibrary.wiley.com/doi/abs/10.1111/j.1532-5415.2005.53221.x .
(33) Fullerton KJ, McSherry D, Stout RW. Albert's test: a neglected test of perceptual neglect. *Lancet (London, England).* 1986; 1 (8478): 430. Available from: https://www.ncbi.nlm.nih.gov/pubmed/2868349 .
(34) EuroQol - a new facility for the measurement of health-related quality of life. *Health policy.* 1990; 16 (3): 199-208. Available from: doi: 10.1016/0168-8510(90)90421-9 Available from: https://www.sciencedirect.com/science/article/pii/0168851090904219 .
(35) NHS England. *The Friends and Family Test.* NHS England. 2014.
(36) SUS: A 'Quick and Dirty' Usability Scale. *Usability Evaluation In Industry.* CRC Press; 1996. pp. 207-212.
(37) Moore GF, Audrey S, Barker M, Bond L, Bonell C, Hardeman W, et al. Process evaluation of complex interventions: Medical Research Council guidance. *BMJ : British Medical Journal.* 2015; 350 (mar19 6): h1258. Available from: doi: 10.1136/bmj.h1258 Available from: http://dx.doi.org/10.1136/bmj.h1258 .
(38) W.K. Kellogg Foundation. *Logic Model Development Guide.* Battle Creek, Michigan: W.K. Kellogg Foundation. 2004.
(39) The Scottish Government. *Designing and Evaluating Behaviour Change Interventions.* The Scottish Government. 2015.
(40) Kislov R, Pope C, Martin GP, Wilson PM. Harnessing the power of theorising in implementation science. *Implementation Science.* 2019; 14 (1): 103. Available from: doi: 10.1186/s13012-019-0957-4 Available from: https://search.proquest.com/docview/2324921962 .
(41) QSR International Ltd. *NVIVO.* Available from: https://www.qsrinternational.com/nvivo/nvivo-products [Accessed 07 Oct 2019].
(42) Braun V, Clarke V. Using thematic analysis in psychology. *Qualitative Research in Psychology.* 2006; 3 (2): 77-101. Available from: doi: 10.1191/1478088706qp063oa Available from: http://www.tandfonline.com/doi/abs/10.1191/1478088706qp063oa .
(43) National Institute for Health Research. *About INVOLVE.* Available from: https://www.invo.org.uk/about-involve/ [Accessed 04 Oct 2019].

For peer review only

Figure 1

Trial diagram

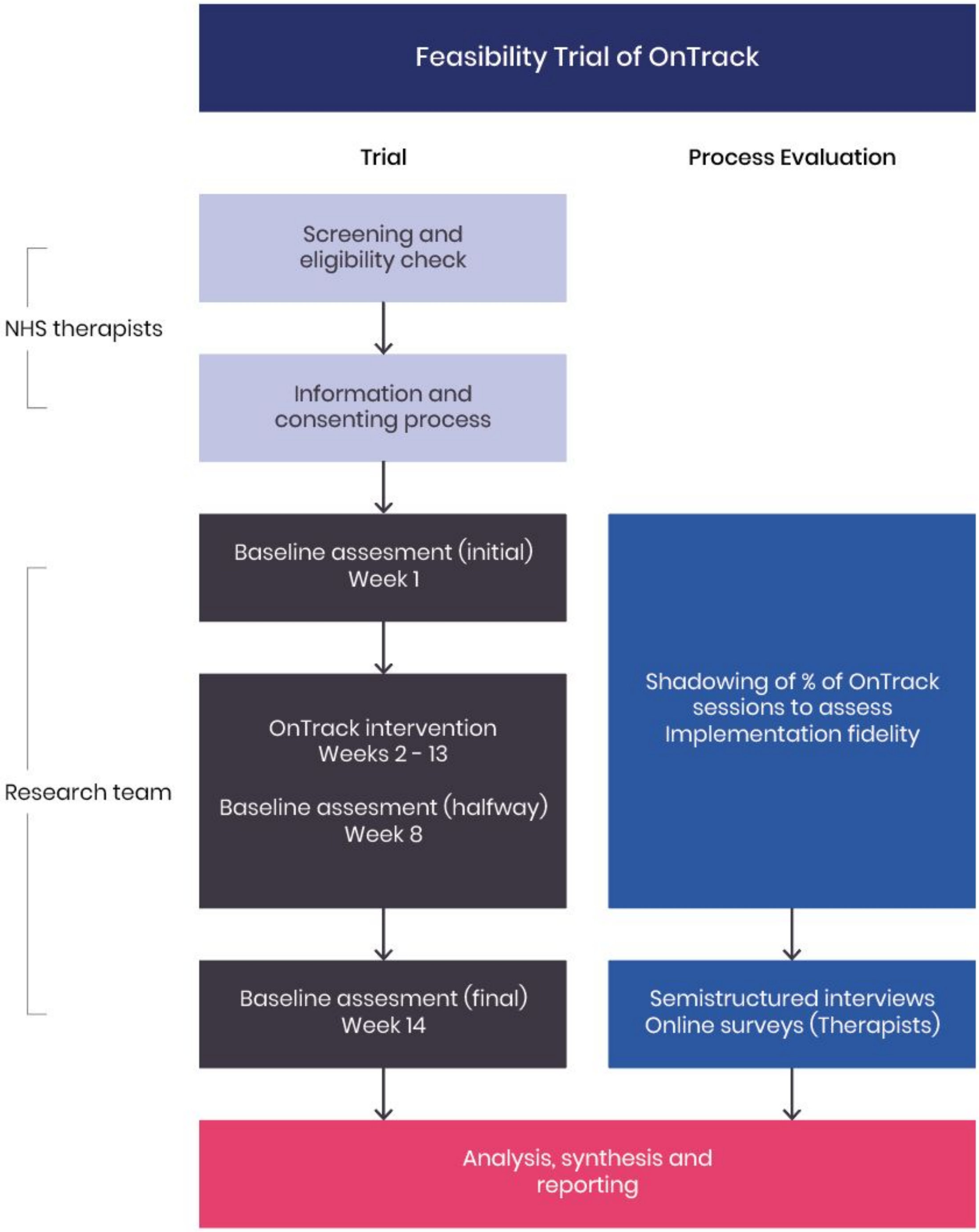

Figure 2

Logic model

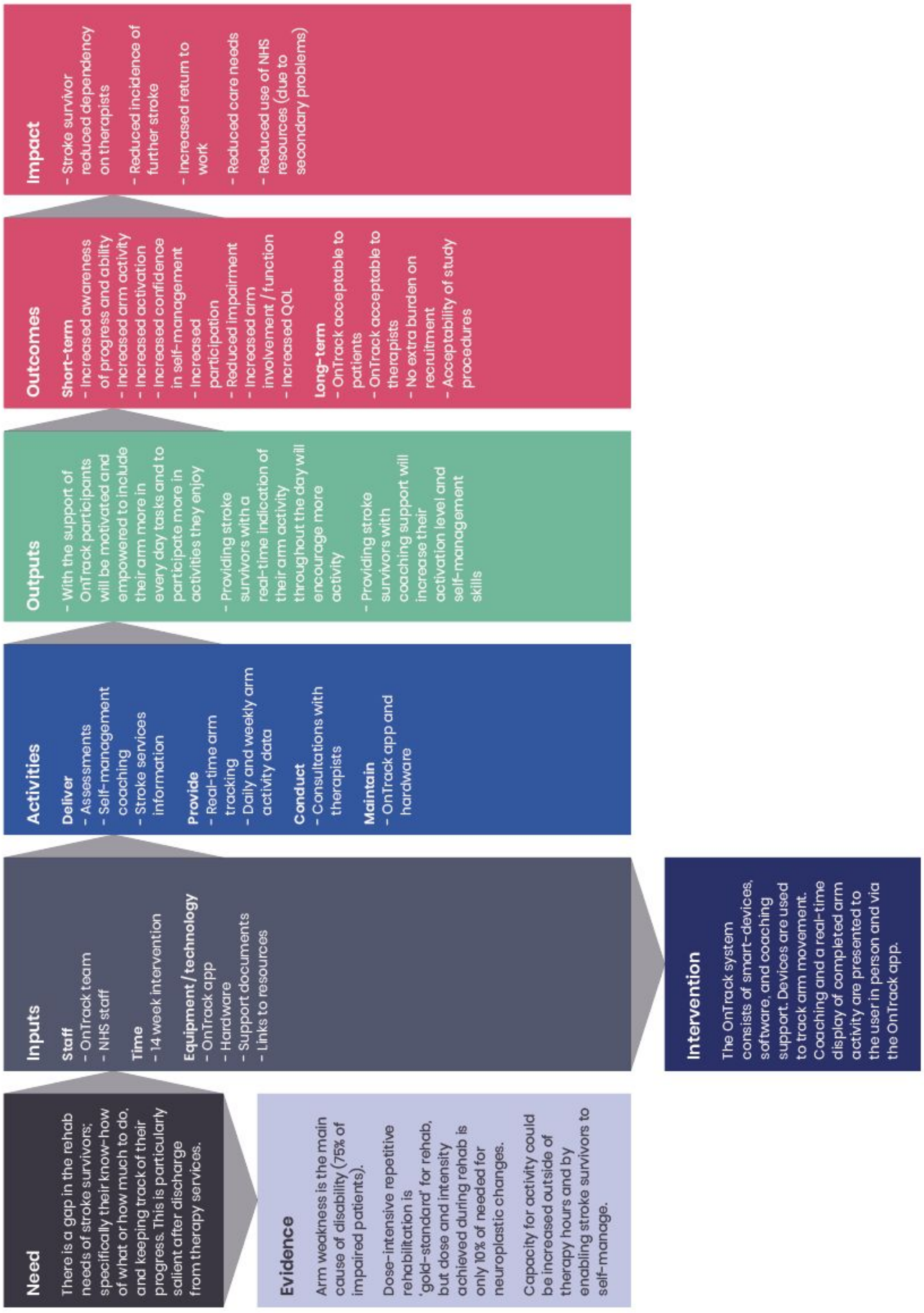

Figure 3

Examples of visualisations created using aggregated data captured by OnTrack from healthy beta testers between June-August 2019

Active minutes per hour of day (0-23) by user, aggregated over time

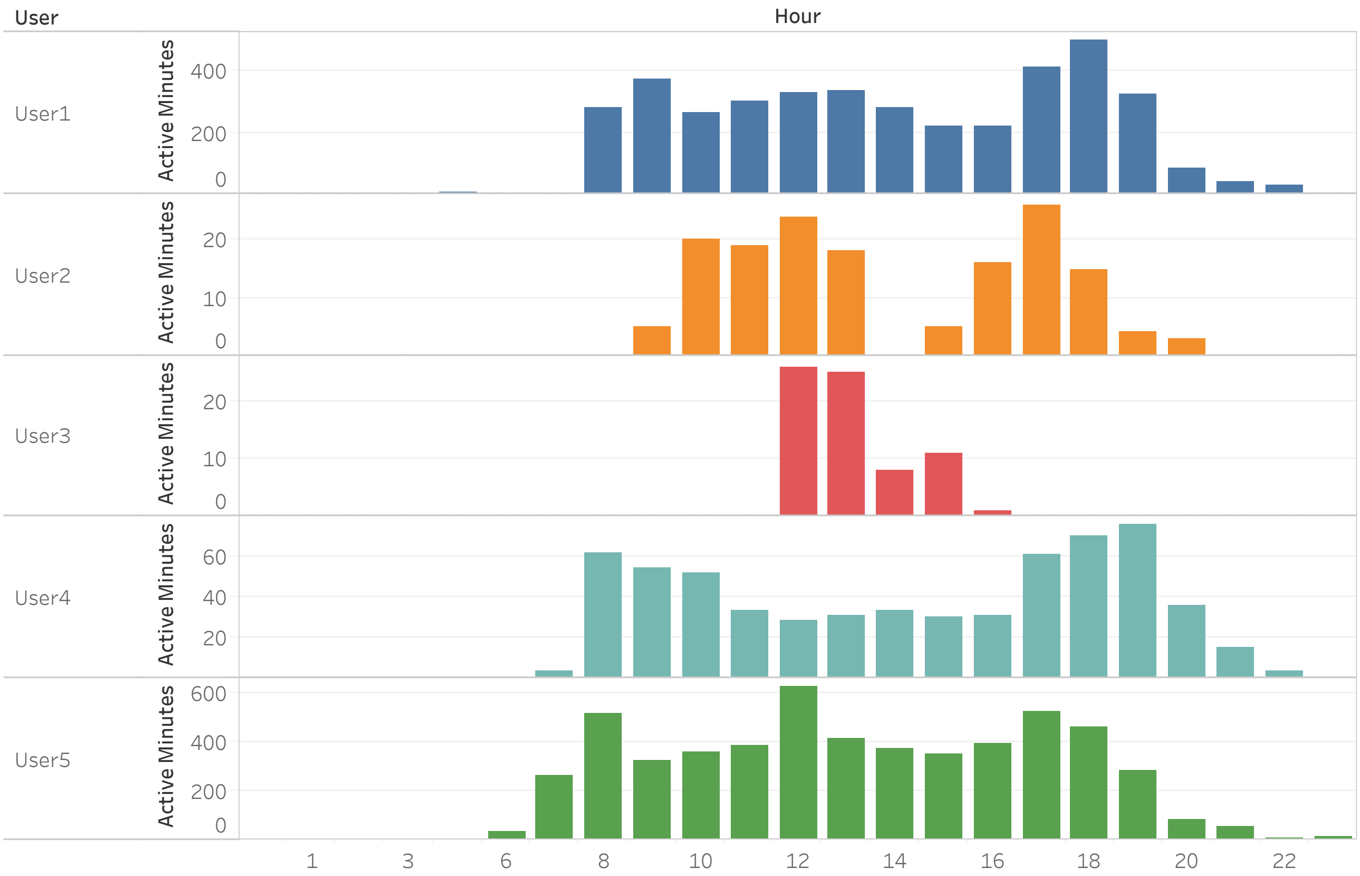

Source: Sample OnTrack usage data collected from healty beta testers, June-August 2019

Active minutes per day by user

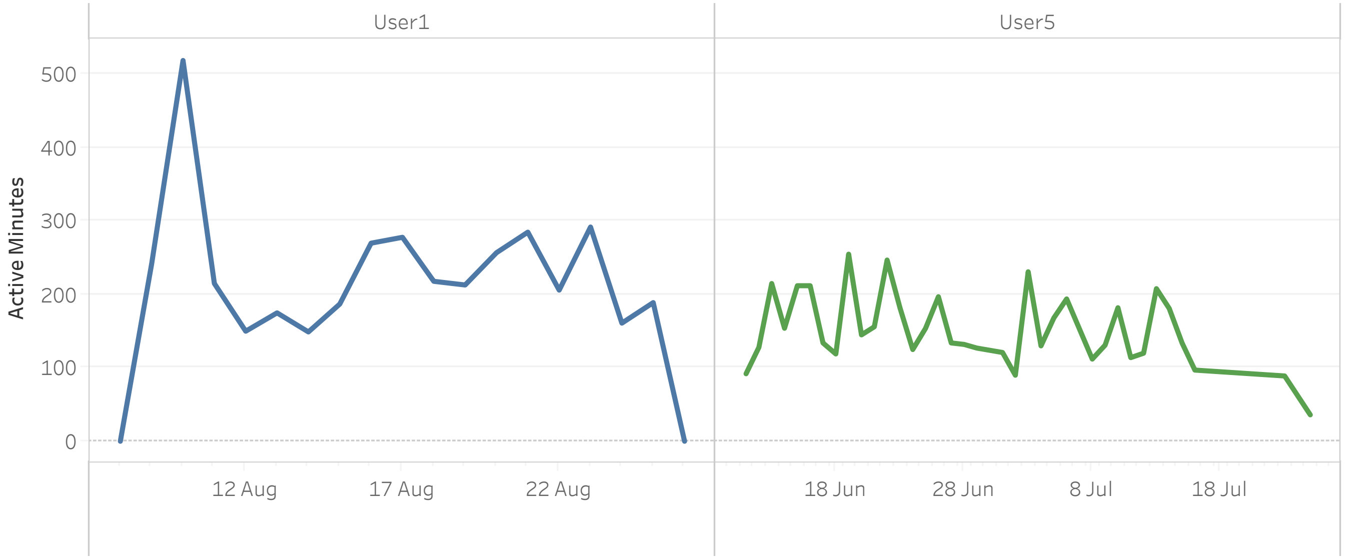

Source: Sample OnTrack usage data collected from healthy beta testers, June-August 2019

Reporting checklist for protocol of a clinical trial.

Based on the SPIRIT guidelines.

Instructions to authors

Complete this checklist by entering the page numbers from your manuscript where readers will find each of the items listed below.

Your article may not currently address all the items on the checklist. Please modify your text to include the missing information. If you are certain that an item does not apply, please write "n/a" and provide a short explanation.

Upload your completed checklist as an extra file when you submit to a journal.

In your methods section, say that you used the SPIRITreporting guidelines, and cite them as:

Chan A-W, Tetzlaff JM, Altman DG, Laupacis A, Gøtzsche PC, Krleža-Jerić K, Hróbjartsson A, Mann H, Dickersin K, Berlin J, Doré C, Parulekar W, Summerskill W, Groves T, Schulz K, Sox H, Rockhold FW, Rennie D, Moher D. SPIRIT 2013 Statement: Defining standard protocol items for clinical trials. Ann Intern Med. 2013;158(3):200-207

		Reporting Item	Page Number
**Administrative information**			
Title	#1	Descriptive title identifying the study design, population, interventions, and, if applicable, trial acronym	1
Trial registration	#2a	Trial identifier and registry name. If not yet registered, name of intended registry	2
Trial registration: data set	#2b	All items from the World Health Organization Trial Registration Data Set	n/a
Protocol version	#3	Date and version identifier	11
Funding	#4	Sources and types of financial, material, and other support	12
Roles and responsibilities: contributorship	#5a	Names, affiliations, and roles of protocol contributors	11

For peer review only

Roles and responsibilities: sponsor contact information	#5b	Name and contact information for the trial sponsor	n/a
Roles and responsibilities: sponsor and funder	#5c	Role of study sponsor and funders, if any, in study design; collection, management, analysis, and interpretation of data; writing of the report; and the decision to submit the report for publication, including whether they will have ultimate authority over any of these activities	11
Roles and responsibilities: committees	#5d	Composition, roles, and responsibilities of the coordinating centre, steering committee, endpoint adjudication committee, data management team, and other individuals or groups overseeing the trial, if applicable (see Item 21a for data monitoring committee)	n/a
**Introduction**			
Background and rationale	#6a	Description of research question and justification for undertaking the trial, including summary of relevant studies (published and unpublished) examining benefits and harms for each intervention	3
Background and rationale: choice of comparators	#6b	Explanation for choice of comparators	n/a
Objectives	#7	Specific objectives or hypotheses	4
Trial design	#8	Description of trial design including type of trial (eg, parallel group, crossover, factorial, single group), allocation ratio, and framework (eg, superiority, equivalence, non-inferiority, exploratory)	5
**Methods: Participants, interventions, and outcomes**			
Study setting	#9	Description of study settings (eg, community clinic, academic hospital) and list of countries where data will be collected. Reference to where list of study sites can be obtained	5
Eligibility criteria	#10	Inclusion and exclusion criteria for participants. If applicable, eligibility criteria for study centres and individuals who will	5

For peer review only

		perform the interventions (eg, surgeons, psychotherapists)	
Interventions: description	#11a	Interventions for each group with sufficient detail to allow replication, including how and when they will be administered	6
Interventions: modifications	#11b	Criteria for discontinuing or modifying allocated interventions for a given trial participant (eg, drug dose change in response to harms, participant request, or improving / worsening disease)	n/a
Interventions: adherance	#11c	Strategies to improve adherence to intervention protocols, and any procedures for monitoring adherence (eg, drug tablet return; laboratory tests)	n/a
Interventions: concomitant care	#11d	Relevant concomitant care and interventions that are permitted or prohibited during the trial	n/a
Outcomes	#12	Primary, secondary, and other outcomes, including the specific measurement variable (eg, systolic blood pressure), analysis metric (eg, change from baseline, final value, time to event), method of aggregation (eg, median, proportion), and time point for each outcome. Explanation of the clinical relevance of chosen efficacy and harm outcomes is strongly recommended	7
Participant timeline	#13	Time schedule of enrolment, interventions (including any run-ins and washouts), assessments, and visits for participants. A schematic diagram is highly recommended (see Figure)	Table 2
Sample size	#14	Estimated number of participants needed to achieve study objectives and how it was determined, including clinical and statistical assumptions supporting any sample size calculations	6
Recruitment	#15	Strategies for achieving adequate participant enrolment to reach target sample size	6
**Methods: Assignment of interventions (for controlled trials)**			
Allocation: sequence generation	#16a	Method of generating the allocation sequence (eg, computer-generated random numbers), and list of any factors for stratification. To reduce predictability of a random sequence, details of any planned restriction (eg, blocking) should be provided in a separate document that is unavailable to those who enrol participants or assign interventions	n/a

For peer review only

Allocation concealment mechanism	#16b	Mechanism of implementing the allocation sequence (eg, central telephone; sequentially numbered, opaque, sealed envelopes), describing any steps to conceal the sequence until interventions are assigned	n/a
Allocation: implementation	#16c	Who will generate the allocation sequence, who will enrol participants, and who will assign participants to interventions	n/a
Blinding (masking)	#17a	Who will be blinded after assignment to interventions (eg, trial participants, care providers, outcome assessors, data analysts), and how	n/a
Blinding (masking): emergency unblinding	#17b	If blinded, circumstances under which unblinding is permissible, and procedure for revealing a participant's allocated intervention during the trial	n/a
**Methods: Data collection, management, and analysis**			
Data collection plan	#18a	Plans for assessment and collection of outcome, baseline, and other trial data, including any related processes to promote data quality (eg, duplicate measurements, training of assessors) and a description of study instruments (eg, questionnaires, laboratory tests) along with their reliability and validity, if known. Reference to where data collection forms can be found, if not in the protocol	9
Data collection plan: retention	#18b	Plans to promote participant retention and complete follow-up, including list of any outcome data to be collected for participants who discontinue or deviate from intervention protocols	n/a
Data management	#19	Plans for data entry, coding, security, and storage, including any related processes to promote data quality (eg, double data entry; range checks for data values). Reference to where details of data management procedures can be found, if not in the protocol	9
Statistics: outcomes	#20a	Statistical methods for analysing primary and secondary outcomes. Reference to where other details of the statistical analysis plan can be found, if not in the protocol	9
Statistics: additional analyses	#20b	Methods for any additional analyses (eg, subgroup and adjusted analyses)	9

For peer review only

Statistics: analysis population and missing data	#20c	Definition of analysis population relating to protocol non-adherence (eg, as randomised analysis), and any statistical methods to handle missing data (eg, multiple imputation)	n/a
**Methods: Monitoring**			
Data monitoring: formal committee	#21a	Composition of data monitoring committee (DMC); summary of its role and reporting structure; statement of whether it is independent from the sponsor and competing interests; and reference to where further details about its charter can be found, if not in the protocol. Alternatively, an explanation of why a DMC is not needed	n/a
Data monitoring: interim analysis	#21b	Description of any interim analyses and stopping guidelines, including who will have access to these interim results and make the final decision to terminate the trial	n/a
Harms	#22	Plans for collecting, assessing, reporting, and managing solicited and spontaneously reported adverse events and other unintended effects of trial interventions or trial conduct	n/a
Auditing	#23	Frequency and procedures for auditing trial conduct, if any, and whether the process will be independent from investigators and the sponsor	n/a
**Ethics and dissemination**			
Research ethics approval	#24	Plans for seeking research ethics committee / institutional review board (REC / IRB) approval	10
Protocol amendments	#25	Plans for communicating important protocol modifications (eg, changes to eligibility criteria, outcomes, analyses) to relevant parties (eg, investigators, REC / IRBs, trial participants, trial registries, journals, regulators)	10
Consent or assent	#26a	Who will obtain informed consent or assent from potential trial participants or authorised surrogates, and how (see Item 32)	6
Consent or assent: ancillary studies	#26b	Additional consent provisions for collection and use of participant data and biological specimens in ancillary studies, if applicable	n/a
Confidentiality	#27	How personal information about potential and enrolled participants will be collected, shared, and maintained in order to protect confidentiality before, during, and after the trial	10

For peer review only

Declaration of interests	#28	Financial and other competing interests for principal investigators for the overall trial and each study site	12
Data access	#29	Statement of who will have access to the final trial dataset, and disclosure of contractual agreements that limit such access for investigators	10
Ancillary and post trial care	#30	Provisions, if any, for ancillary and post-trial care, and for compensation to those who suffer harm from trial participation	n/a
Dissemination policy: trial results	#31a	Plans for investigators and sponsor to communicate trial results to participants, healthcare professionals, the public, and other relevant groups (eg, via publication, reporting in results databases, or other data sharing arrangements), including any publication restrictions	10
Dissemination policy: authorship	#31b	Authorship eligibility guidelines and any intended use of professional writers	n/a
Dissemination policy: reproducible research	#31c	Plans, if any, for granting public access to the full protocol, participant-level dataset, and statistical code	n/a
**Appendices**			
Informed consent materials	#32	Model consent form and other related documentation given to participants and authorised surrogates	n/a
Biological specimens	#33	Plans for collection, laboratory evaluation, and storage of biological specimens for genetic or molecular analysis in the current trial and for future use in ancillary studies, if applicable	n/a

The SPIRIT checklist is distributed under the terms of the Creative Commons Attribution License CC-BY-ND 3.0. This checklist was completed on 07. October 2019 using https://www.goodreports.org/, a tool made by the EQUATOR Network in collaboration with Penelope.ai

For peer review only
